# Biomimetic Enzymatic Oxidative Coupling of Barley Phenolamides: Hydroxycinnamoylagmatines

**DOI:** 10.1021/acs.jafc.2c07457

**Published:** 2022-12-14

**Authors:** Annemiek van Zadelhoff, Lieke Meijvogel, Anna-Marie Seelen, Wouter J.C. de Bruijn, Jean-Paul Vincken

**Affiliations:** Laboratory of Food Chemistry, Wageningen University & Research, Bornse Weilanden 9, WG Wageningen 6708, Netherlands

**Keywords:** hordatine, (neo) lignanamide, murinamide, hydroxycinnamic acid amides, NMR, LC−MS

## Abstract

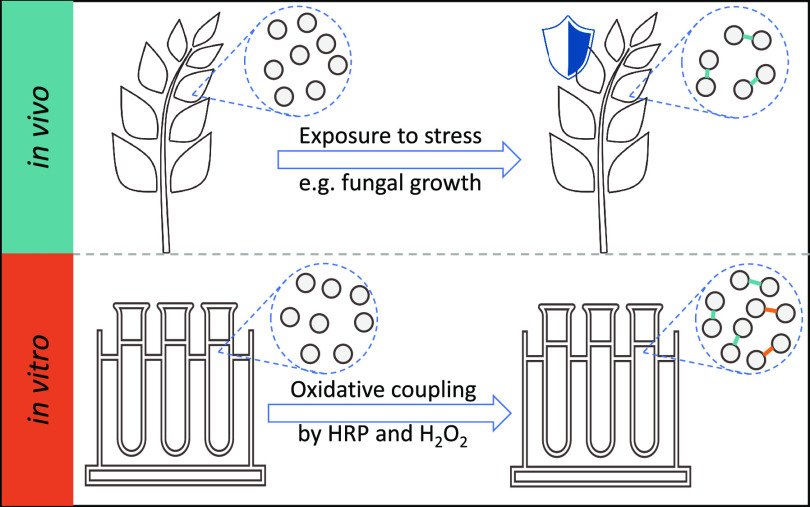

Oxidative coupling of hydroxycinnamoylagmatines in barley (*Hordeum vulgare*) and related *Hordeum* species is part of the plant defense mechanism. Three linkage types have been reported for hydroxycinnamoylagmatine dimers, but knowledge on oxidative coupling reactions underlying their formation is limited. In this study, the monomers coumaroylagmatine, feruloylagmatine, and sinapoylagmatine were each incubated with horseradish peroxidase. Their coupling reactivity was in line with the order of peak potentials measured: sinapoylagmatine (245 mV) > feruloylagmatine (341 mV) > coumaroylagmatine (506 mV). Structure elucidation of fourteen *in vitro* coupling products by NMR and MS revealed that the three main linkage types were identical to those naturally present in *Hordeum* species, namely, 4-*O*-7′/3-8′, 2-7′/8-8′, and 8-8′/9-*N*-7′. Furthermore, we identified two linkage types that were not previously reported for hydroxycinnamoylagmatine dimers, namely, 8-8′ and 4-*O*-8′. We conclude that oxidative coupling by horseradish peroxidase can be used for biomimetic formation of natural antifungal hydroxycinnamoylagmatine dimers from barley.

## 1 Introduction

**Figure 1:**
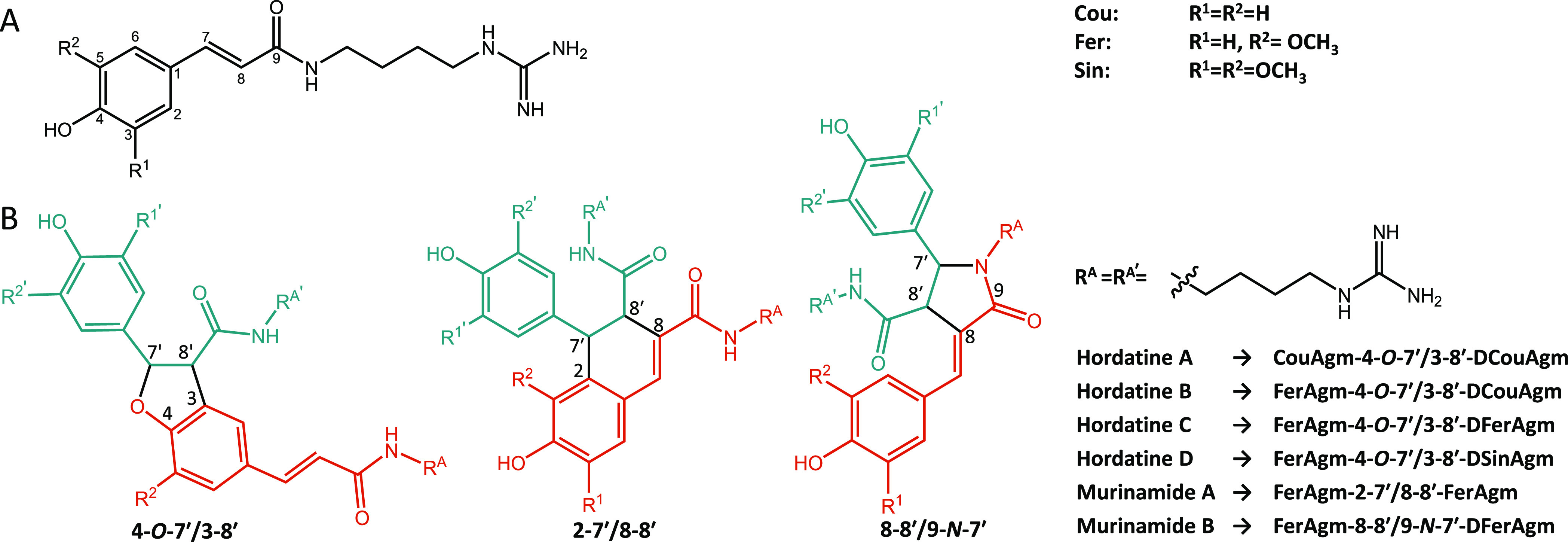
General structure of hydroxycinnamoylagmatines (A) and the structure of the three previously reported hydroxycinnamoylagmatine linkage types (B), namely, 4-O-7′/3-8′, 2-7′/8-8′, and 8-8′/9-N-7′ with their trivial and systematic names.^1−3,10,16^

Barley (*Hordeum vulgare*) produces hydroxycinnamoylagmatines (HCAgms) as secondary metabolites that protect the plant against environmental and biological threats.^1−3^ These compounds are found in barley seeds and malted barley and thus also end up in barley-derived food products, such as beer.^4^ HCAgms belong to the group of phenolamides, also known as hydroxycinnamic acid amides, and are composed of a hydroxycinnamic acid moiety linked to an agmatine group by an amide bond^1^ (Figure 1A). Hydroxycinnamic acids naturally present in barley HCAgms are coumaric acid, ferulic acid, and sinapic acid.^4^ Upon exposure to stress, such as high or low temperatures or fungal infection, the concentration of HCAgms in barley was increased more than 10-fold.^5−7^ Apart from an increase in hydroxycinnamoylagmatines, the concentration of dimeric compounds and the activity of peroxidase, which has been proposed to be involved in the formation of dimers, was also reported to increase upon exposure to fungal stress.^1,8,9^ This indicates that HCAgms play an important role in the plant defense mechanism and their dimerization via oxidative coupling might be essential in the plant’s defense against fungi.

The three types of dimeric compounds that have been identified for hydroxycinnamoylagmatines are hordatines in barley^1−3^ and two types of murinamides in related *Hordeum* species, that is, *H. murinum* and *H. bulbosium*(10) (Figure 1B). The different types of dimeric coupling products of HCAgmMs are active as antifungals against several species of fungi.^7,8,10−14^ Additionally, coupling products of other phenolamides have been reported to possess a large variety of bioactivities, such as anticarcinogenic and anti-inflammatory activities.^15^ However, the bioactivity of HCAgm coupling products has not been studied in detail, possibly due to the lack of commercial standards and the fact that purification from barley is laborious. These HCAgm dimers belong to the class of (neo)lignanamides, which consists of diverse phenolamide coupling products containing many different linkage types and higher degrees of polymerization (i.e., trimers).^16^ Thus, due to their structural similarities with other phenolamides, it is expected that HCAgms are also able to form a larger variety of linkage types and products with a higher degree of polymerization upon in vitro oxidative coupling.

In this study, a nomenclature for (neo)lignanamides^16^ will be used to simplify translation of the dimer name to the monomer composition and linkage type of the dimers. The main focus in the literature is on CouAgm-4-*O*-7′/3-8′-DCouAgm (also known as hordatine A) and FerAgm-4-*O*-7′/3-8′-DCouAgm (also known as hordatine B).^2,3,11^ Fewer studies focus on the formation and content of FerAgm-4-*O*-7′/3-8′-DFerAgm (also known as hordatine C) and FerAgm-4-*O*-7′/3-8′-DSinAgm (also known as hordatine D).^4,17^ In some cases, hordatine C is suggested to be a coumaroylagmatine and sinapoylagmatine heterodimer.^3^ The two types of murinamides will be referred to as FerAgm-2-7′/8-8′-DFerAgm and FerAgm-8-8′/9-*N*-7′-DFerAgm.

Dimers formed by the three currently known linkage types are structural isomers, so a simultaneous formation of these three linkage types and perhaps even additional ones result in a complex mixture of dimeric compounds with the same molecular mass. Moreover, the possible structural variety can be even further increased due to possible combinations of three different precursors. Additionally, each monomer and most of the reported dimers also possess at least one double bond, which can undergo isomerization from *trans* to *cis*, for example, under the influence of light or temperature.^18^ Moreover, all reported HCAgm coupling products contain two chiral carbons (i.e., C7′ and C8′). Due to the lack of mass spectral information for HCAgm coupling products, identification of these compounds in mixtures by LC–MS is challenging. Therefore, studying naturally occurring HCAgm dimers in a mixture is complicated and the reactions underlying the formation of dimers with various linkage types by peroxidase is poorly understood. To better understand the effect of substitution of the hydroxycinnamic acid moiety on the stability and reactivity of the HCAgm monomers and to gain insight in the type of coupling products formed from HCAgms, we will study these compounds in model systems using horseradish peroxidase (HRP) since the in vivo coupling is also suggested to be catalyzed by a peroxidase.^2^ To this end, we aim to develop an approach for the biomimetic synthesis of these coupling products, which will be a useful tool to facilitate establishing structure–reactivity relationships of the HCAgm monomers, understanding the formation of natural HCAgm coupling products, and studying the bioactivity of these coupling products. Furthermore, the aim is to establish the MS spectral properties of the coupling products to enable their identification in more complex mixtures, such as barley-derived food products. Oxidative coupling of hydroxycinnamoylagmatines by HRP is expected to result in the formation of the linkage types found in barley and related *Hordeum* species as well as linkage types known for other (neo)lignanamides. This will be studied by analyzing the coupling products using reversed-phase ultrahigh performance liquid chromatography combined with photodiode array detection and ion trap mass spectrometry (RP-UHPLC-PDA-IT-MS), high-resolution Orbitrap mass spectrometry (RP-UHPLC-PDA-FT-MS), nuclear magnetic resonance (NMR) spectroscopy, and matrix-assisted laser desorption/ionization time-of-flight mass spectrometry (MALDI-TOF-MS).

## 2 Material and Methods

### 2.1 Materials

*p*-Coumaric acid (98%), sinapic acid (98%), ferulic acid (99%), *N*,*N*′-dicyclohexylcarbodiimide (DCC), sodium bicarbonate (NaHCO_3_), sodium hydroxide, 2,2′-azino-bis(3-ethylbenzothiazoline-6-sulfonic acid) (ABTS), nitric acid, horseradish peroxidase type VI-A (HRP, 1.11.1.7, P6782), deuterated methanol, and 30% (*w/w*) hydrogen peroxide were purchased from Sigma-Aldrich (St. Louis, MO, USA). Agmatine sulfate (97%) was purchased from Fisher Scientific B.V. (Hampton, New Hampshire, USA), and 2,5-dihydroxybenzoic acid was obtained from Bruker Daltonics (Bremen, Germany). Ammonium acetate, potassium dihydrogenphosphate, and dipotassium hydrogen phosphate were purchased from Merck KGaA (Darmstadt, Germany). Glacial acetic acid was purchased from VWR International BV (Radnor, PA, USA). Ethyl acetate, acetone, methanol (MeOH), hexane, HPLC grade acetonitrile (ACN), UHPLC–MS grade ACN and water, and formic acid (FA) (99% [*v*/*v*]) were purchased from Biosolve (Valkenswaard, Netherlands). Water for purposes other than UHPLC was purified using a Milli-Q purification system equipped with a 0.22 μm filter (Millipore, Molsheim, France).

### 2.2 Synthesis of Hydroxycinnamoylagmatine Monomers

*p*-Coumaroylagmatine (CouAgm), feruloylagmatine (FerAgm), and sinapoylagmatine (SinAgm) were synthesized according to the protocol described by van Zadelhoff et al.^19^ In short, equimolar amounts of the individual hydroxycinnamic acids and *N*,*N*′-dicyclohexylcarbodiimide(DCC) in acetone were stirred for 1 h at room temperature in the dark. After 1 h, an equimolar amount of agmatine sulfate and sodium bicarbonate dissolved in water in a volume equal to the acetone was added. The mixture was stirred for 24 h at room temperature in the dark, and after which, the reaction was stopped by adding an equimolar amount of acetic acid. The synthesized HCAgms were purified by flash chromatography using a 12 g FlashPure ID C18 column (Büchi). The eluents used were water with 1% (*v*/*v*) FA (eluent A) and ACN with 1% (*v*/*v*) FA (eluent B). Fractions were collected based on the absorbance at 290, 300, 310, and 320 nm. The elution program used was 2 column volumes (CVs) isocratic at 5% B, 20 CVs linear gradient to 25% B, 1 CV linear gradient to 100% B, and 5 CVs isocratic at 100% B. After collecting and pooling the fractions containing the desired product, ACN was evaporated under reduced pressure at 40 °C. The remaining water was removed by freeze-drying.

### 2.3 Thermal and Light Stability of Hydroxycinnamoylagmatine Monomers

A mixture of CouAgm, FerAgm, and SinAgm dissolved in 50% (*v*/*v*) aqueous ACN (0.1 mM each monomer) was prepared. To investigate the light stability, eight tubes containing the mixture were exposed to laboratory light at room temperature. After 0, 1, 3, and 7 days, two tubes were covered with aluminum foil and stored at room temperature in the dark. To investigate the temperature stability of the monomers, different tubes containing the mixture were incubated at either 20, 50, or 90 °C for 10 and 60 min in the dark. Furthermore, the mixture was incubated at 90 °C for 10 and 60 min in the presence of laboratory light. For both experiments, the amount of monomers and the conversion of the *trans* isomer into its *cis* isomer were monitored by RP-UHPLC-PDA-FT-MS.

### 2.4 HRP Activity Determination

The activity of HRP was measured using ABTS, according to the method described by Heijnis et al.^20^ The activity of HRP in a 0.1 M ammonium acetate buffer pH 7.0 was found to be 186.8 U (1 U = 1 μmol ABTS oxidized per min per mg HRP) at 30 °C (Figure S1).

### 2.5 Enzymatic Oxidative Coupling of Hydroxycinnamoylagmatines

#### 2.5.1 Small-Scale Oxidative Coupling

To study the optimal reaction conditions and the reactivity of the different hydroxycinnamoylagmatine (HCAgm) monomers, oxidative coupling using HRP and H_2_O_2_ was performed on a small scale. CouAgm, FerAgm, or SinAgm was separately dissolved in 50% (*v*/*v*) aqueous ACN to a concentration of 0.05 M. The standard conditions for small-scale oxidative coupling were as follows: the monomer solution (400 μL) was mixed with a 0.1 M ammonium acetate buffer at pH 7 (2 mL) and a 5 μg/mL HRP solution (187 μL). The mixture was equilibrated in a thermomixer (Eppendorf, Hamburg, Germany) at 30 °C and 500 rpm for 5 min. To start the reaction, H_2_O_2_ (20 μL, 0.3% (*v*/*v*)) was added. The mixture was incubated in a thermomixer at 30 °C and 500 rpm and samples (10 μL) were taken at 0, 15, 30, 60, and 120 min. The samples were diluted 100 times in water and heated at 90 °C and 500 rpm for 3 min in a thermomixer to inactivate the HRP. After taking samples at 30 and 60 min, H_2_O_2_ solution (20 μL, 0.3% (*v*/*v*)) was added to the reaction mixture, which should result in restoring the original H_2_O_2_ concentration, assuming that all previously added H_2_O_2_ had reacted. To check for pH dependency of the reaction, additional incubations were performed for CouAgm in ammonium acetate buffers of pH 5 and 8.5 with all other conditions retained as mentioned above.

For all incubations, substrate blanks with H_2_O_2_ but without HRP were prepared. All samples were prepared in triplicate and were analyzed by RP-UHPLC-PDA-IT-MS and RP-UHPLC-PDA-FT-MS. Prior to analysis, the samples were centrifuged (16,000 × *g*, 5 min).

#### 2.5.2 Large-Scale Oxidative Coupling

For the purification of the coupling products, oxidative coupling was performed on a larger scale. The protocol and all concentrations and ratios used were the same as for the small-scale incubations; however, the total volume used was 6 mL of monomer solution in 30 mL of ammonium acetate buffer at pH 7. The samples were incubated in an oven equipped with a head-over-tail rotator. After 120 min the enzyme was inactivated by placing the samples in a 90 °C water bath for 10 min. Samples of the reaction product were diluted 100 times and centrifuged (16,000 × *g*, 5 min) prior to analysis by RP-UHPLC-PDA-IT-MS.

### 2.6 Purification of Coupling Products by Preparative Chromatography

The large-scale oxidative coupling samples were used for purification of the coupling products. Prior to purification, the samples were concentrated under reduced pressure at 60 °C to a volume less than 24 mL in order to remove ACN from the sample. Purification was performed using fraction collection after separation by reversed-phase flash chromatography on a Pure C-850 FlashPrep system (Büchi, Flawil, Switzerland) operated in the flash mode using liquid loading. Separation was performed on a 12 g FlashPure ID C18 column (Büchi). The eluents used were water with 1% (*v*/*v*) FA (eluent A) and ACN with 1% (*v*/*v*) FA (eluent B). Fractions were collected using the collect-all mode. The elution program used was 1 column volume(CV) isocratic at 0% B, 50 CVs linear gradient to 25% B, 1 CV linear gradient to 100% B, and 5 CVs isocratic at 100% B. All fractions collected were analyzed by RP-UHPLC-PDA-IT-MS. Fractions were combined into pools containing two or three compounds. After collecting and pooling the fractions, ACN was evaporated under reduced pressure at 40 °C. The remaining water was removed by freeze-drying.

The obtained pools were further purified by using the Pure C-850 FlashPrep system in the prep mode. The freeze-dried pools were redissolved in the smallest amount of water possible, and per prep run, a maximum sample volume of 1 mL was loaded on to an XBridge Prep C18 OBD column (250 mm × 19 mm i.d., 5 μm particle size) (Waters, Milford, MA, USA). The eluents used were water with 1% (*v*/*v*) FA (eluent A) and ACN with 1% (*v*/*v*) FA (eluent B). Fractions were collected using the collect-all mode. The elution profiles used were optimized for each fraction and are reported in the Supporting Information. All fractions collected were analyzed by RP-UHPLC-PDA-IT-MS. Fractions were combined into pools containing one pure compound. After collecting and pooling the fractions, ACN was evaporated under reduced pressure at 40 °C. The remaining water was removed by freeze-drying.

### 2.7 Monitoring Monomer Reactivity and Profiling Reaction Products by RP-UHPLC-PDA-IT-MS Analysis

For analysis of hydroxycinnamoylagmatines and their coupling products, the samples were separated on a Thermo Vanquish UHPLC system (Thermo Scientific, San Jose, CA) equipped with a pump, degasser, autosampler, and PDA detector. The analytical method was optimized for the analysis of the HCAgm monomers and dimers by reversed-phase ultrahigh performance liquid chromatography combined with photodiode array detection and ion trap mass spectrometry (RP-UHPLC-PDA-IT-MS). Different column temperatures (25, 35, and 45 °C), acid concentrations in the eluents (0.1 and 1% FA), and slopes of the gradient (0.5, 1, and 2% B per column volume) were investigated (data not shown). As the hydroxycinnamoylagmatines ionized better in the positive ionization mode, only the positive ionization mode was used in the optimized method. The conditions of the optimized method are described below and were used unless stated otherwise.

The flow rate was set at 400 μL/min at a column temperature of 35 °C. The PDA detector was set to measure wavelengths in the range of 190–680 nm. Water (A) and ACN (B), both acidified with 1% (*v*/*v*) formic acid, were used as eluents. Samples (1 μL) were injected on a Waters Acquity BEH C18 column (150 mm × 2.1 mm i.d., 1.7 μm particle size) with a VanGuard guard column of the same material (5 mm × 2.1 mm i.d., 1.7 μm particle size) (Waters, Milford, MA, USA). The following elution program was used: isocratic at 5% B for 1.10 min, 1.10–34.02 min linear gradient to 20% B, 34.02–35.12 min linear gradient to 100% B, and 35.12–40.61 min isocratic at 100% B. The eluent was adjusted to its starting composition in 1.10 min followed by equilibration for 5.49 min.

Mass spectrometric data were acquired using a Velos Pro linear ion trap mass spectrometer (Thermo Scientific, San Jose, CA, USA) equipped with a heated ESI probe coupled in-line to the Vanquish RP-UHPLC system. Nitrogen was used as sheath gas (50 arbitrary units) and auxiliary gas (13 arbitrary units). The source conditions were a capillary temperature of 263 °C, a probe heater temperature of 425 °C, and a source voltage of 3.5 kV. The S-Lens RF level was 67.63%. Data was collected in the positive ionization mode over the *m*/*z* range 200–1500. Fragmentation of the most abundant ions in full MS was performed by collision-induced dissociation (CID) with a normalized collision energy of 35%. Dynamic exclusion with a repeat count of 3, a repeat duration of 5.0 s, and an exclusion duration of 10.0 s was used to obtain MS^2^ spectra of multiple different ions present in full MS at the same time. Most settings were optimized via automatic tuning using LTQ Tune Plus (Xcalibur version 4.1, Thermo Scientific). Data processing was performed using Xcalibur 4.1 (Thermo Scientific).

### 2.8 Accurate Mass Determination by RP-UHPLC-PDA-FT-MS Analysis

To accurately determine the mass of the hydroxycinnamoylagmatines, their coupling products, and the fragments formed, the samples were analyzed with RP-UHPLC-PDA and high-resolution Orbitrap mass spectrometry (FT-MS). The samples were separated on a Vanquish RP-UHPLC system (Thermo Scientific, San Jose, CA). The injection volume was 1 μL. The column, mobile phases, and elution program were identical to those described for RP-UHPLC-PDA-IT-MS analysis.

Accurate mass spectrometric data were acquired using a Thermo Q Exactive Focus hybrid quadrupole-Orbitrap mass spectrometer (Thermo Scientific, San Jose, CA) equipped with a heated ESI probe coupled in-line to the Vanquish RP-UHPLC system. Full MS data were collected over an *m*/*z* range of 250–800 in PI. Prior to analysis, the mass spectrometer was calibrated in PI using Tune 2.9 software (Thermo Scientific) by injection of a Pierce positive ion calibration solution (Thermo Scientific). Nitrogen was used as a sheath gas (50 arbitrary units) and auxiliary gas (13 arbitrary units). The source conditions were a capillary temperature of 263 °C, a probe heater temperature of 425 °C, and a source voltage of 3.5 kV. The S-Lens RF level was 50%. Fragmentation was performed by higher-energy collisional dissociation (HCD) with a normalized collision energy of 15%. Full MS and MS^2^ data were recorded at 70,000 and 17,500 resolutions, respectively. Data processing was performed using Xcalibur 4.1 (Thermo Scientific).

### 2.9 Structure Elucidation by Nuclear Magnetic Resonance (NMR) Spectroscopy

For the structure elucidation of the monomers and coupling products, between 1.5 and 2.5 mg of the compound was dissolved in 500 μL of deuterated methanol (Sigma-Aldrich). NMR spectra were recorded at a probe temperature of 300 K on a Bruker Avance-III-600 spectrometer with a cryoprobe (Bruker, Billerica, MA, USA) located at the Magnetic Resonance Research Facility of Wageningen University. For all compounds, 1D ^1^H and ^13^C and 2D HMBC and HMQC spectra were acquired. Data processing was performed using TopSpin 4.1.1 (Bruker).

### 2.10 Peak Potential Measurement by Cyclic Voltammetry

Cyclic voltammograms were obtained by adding 200 μL of a 20 mM stock solution of one of the HCAgms in ethanol to 20 mL of the supporting electrolyte, a 0.1 M phosphate buffer at pH 7.3, in a one-compartment three-electrode cell. Voltammograms were recorded at room temperature using a glassy carbon working electrode, a platinum counter electrode, and an Ag/AgCl saturated KCl reference electrode. The glassy carbon probe was polished before each measurement. A pH meter with a glass electrode was used for the pH measurements. During the measurement, the sample was flushed with N_2_. The peak potentials were determined using the first derivative of the cyclic voltammograms in which the peak potential is equal to the *x* value corresponding to *y* = 0 or, if *y* = 0 does not exist, the *x* value corresponding to the *y* value minimum after the first peak.

### 2.11 Oligomer Analysis by MALDI-TOF-MS

The formation of larger oligomers upon enzymatic oxidative coupling (*t* = 120 min) was investigated using matrix-assisted laser desorption/ionization time-of-flight mass spectrometry (MALDI-TOF-MS). Untreated monomer solutions and substrate blank solutions (*t* = 0) were also analyzed. 2,5-Dihydroxybenzoic acid was used as a matrix. Spots were prepared by mixing 1 μL of the matrix solution with 1 μL of the sample on a MTP 384 ground steel target plate (Bruker Daltonics). Mass spectra (*m*/*z* 260–3000 for the untreated solutions and 500–3000 for the treated solutions) were obtained in the positive ionization mode using an ultrafleXtreme workstation equipped with a Smartbeam-II laser of 355 nm controlled by FlexControl 3.4 software (Bruker Daltonics). Spectra were collected at a laser intensity of 35% with an ion source voltage of 20.00 kV. The frequency of the laser was 1000 Hz. The system was calibrated using maltodextrin. All samples were analyzed in duplicate. Data processing was performed using FlexAnalysis 3.4 (Bruker Daltonics).

## 3 Results and Discussion

### 3.1 Thermal and Light Stability of Hydroxycinnamoylagmatines

Prior to studying their oxidative coupling, the thermal and light stability of the three hydroxycinnamoylagmatine (HCAgm) monomers used in this study were tested. Data for the thermal and light stability is given in Figures S2 and S3. For all three HCAgms, no *trans*–*cis* isomerization was observed upon heating for 60 min at 20 and 50 °C in the dark and upon heating at 90 °C both with and without light exposure. These results showed that the monomers can be safely exposed to elevated temperatures, for example, during the thermal enzyme inactivation step without the risk of chemical conversion of the monomer. The light stability of the HCAgms was tested at room temperature for seven days. For all monomers, conversion from the *trans* to the *cis* isomer was observed. Sinapoylagmatine (SinAgm) and feruloylagmatine (FerAgm) showed the highest susceptibility to isomerization with 24% of the *trans* isomer remaining after three days of incubation. Coumaroylagmatine (CouAgm) was less sensitive to photoisomerization with 31% of the *trans* isomer remaining after seven days. The susceptibility of the different HCAgms to photoisomerization is in line with reports on the light stability of the corresponding hydroxycinnamic acids in which sinapic acid and ferulic acid were also found to be more susceptible to photo-oxidation upon exposure to UV light than *p*-coumaric acid.^21^

### 3.2 Enzymatic Oxidative Coupling Reactivity of Hydroxycinnamoylagmatines

**Figure 2:**
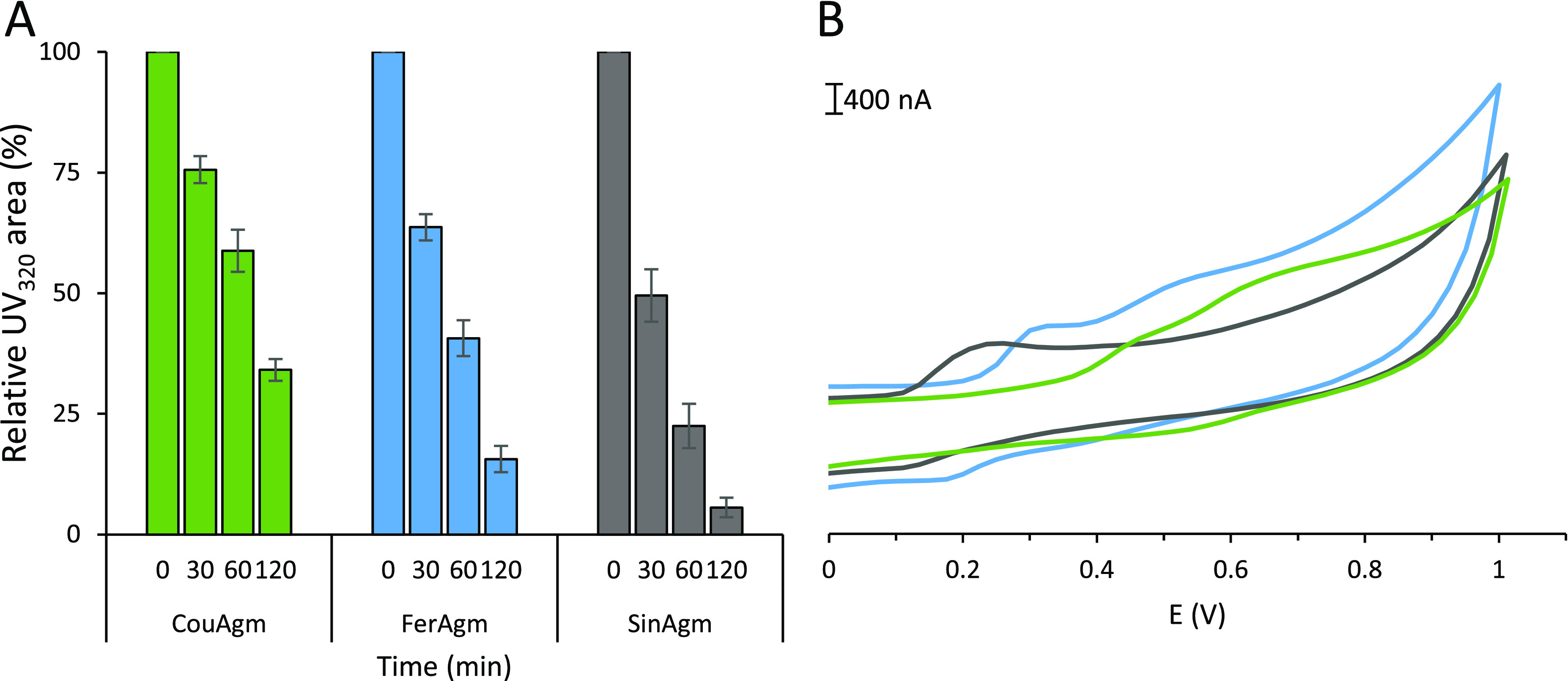
Percentage of remaining monomer upon oxidative coupling of coumaroylagmatine (CouAgm), feruloylagmatine (FerAgm), and sinapoylagmatine (SinAgm) with horseradish peroxidase and H_2_O_2_ expressed as the relative UV_320_ peak area of the corresponding peak in RP-UHPLC-PDA-IT-MS (A). Error bars display the standard deviation based on triplicates. Cyclic voltammograms (B) for 0.2 mM CouAgm (green), FerAgm (blue), and SinAgm (gray) with a glassy carbon electrode in pH 7.3 phosphate buffer.

The effect of phenolic ring substitution on the oxidative coupling reactivity was investigated by individually incubating the HCAgms with horseradish peroxidase (HRP) for 2 h. The reactivity of SinAgm was the highest, whereas the reactivity of CouAgm was the lowest, with monomer conversions of 94.4% (SD 2.1%) and 65.9% (SD 2.3%), respectively. For FerAgm, 84.4% (SD 2.6%) of the monomer was converted after 120 min (Figure 2A). No previous reports are available on the redox or peak potentials of HCAgms; therefore, these were determined using cyclic voltammetry (Figure 2B). As the cyclic voltammograms showed irreversible behavior, no redox potentials could be determined, and therefore, the peak potentials (*E*_P_) were determined instead, resulting in an *E*_p_ of 245 mV (SD 3 mV) for SinAgm, 341 mV (SD 3 mV) for FerAgm, and 506 mV (SD 5 mV) for CouAgm. These potentials follow the same order as those reported for the hydroxycinnamic acids that constitute our HCAgms, namely, the peak potentials are lowered by increasing ring substitution.^22−24^ The lower reactivity of CouAgm can be explained by the higher peak potential compared to FerAgm and SinAgm. The relative reactivity of the hydroxycinnamoylagmatines is in line with the reactivity of the corresponding hydroxycinnamic acids, indicating that the agmatine moiety does not impact the relative reactivity of the monomers.

### 3.3 Identification of Purified Coupling Products

**Figure 3:**
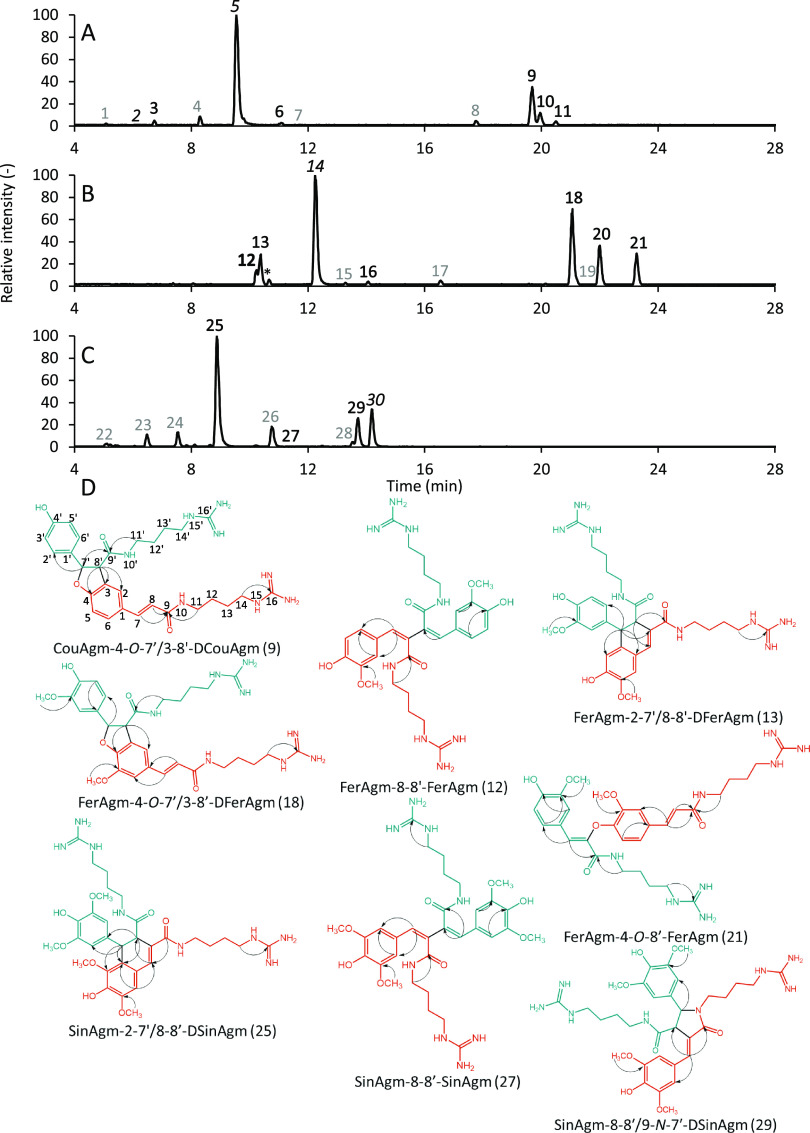
RP-UHPLC-PDA-IT-MS base peak chromatograms (*m*/*z* 250–1500) in the positive ionization mode of CouAgm (A), FerAgm (B), and SinAgm(C) after 120 min of small-scale incubation with HRP. Peak numbers indicate monomers (black, italics), identified coupling products (black, bold), and unidentified compounds (gray). * indicates an impurity present since the beginning of the reaction, which was not converted during the oxidative coupling reaction. Key HMBC correlations (D) for compounds 9, 12, 13, 18, 21, 25, 27, and 29. Monomeric constituents are shown in orange and blue.

The chromatograms obtained after 120 min of incubation of the individual hydroxycinnamoylagmatines with HRP and H_2_O_2_ are shown in Figure 3. Based on the RP-UHPLC-MS data, a large variety of coupling products was formed upon oxidative coupling by HRP. Even though only three different linkage types have previously been reported for coupling products of hydroxycinnamoylagmatines,^3,10^ comparison of fragmentation patterns among the peaks observed in our reaction mixtures hints at the formation of dimers with more than three different linkage types. However, direct identification of linkage types based on fragmentation is not possible, as fragmentation data is not available from the literature. It is, therefore, of great interest to isolate and identify the coupling products and to establish the structure of potentially new linkage types. To this end, the coupling reaction was also performed on a larger scale. Large-scale oxidative coupling resulted in a lower monomer conversion; however, the coupling products formed were the same (Figure S4). The reaction products were separated into pools containing two or three products using flash chromatography (Figure S5). These pools were further purified using preparative chromatography, resulting in the purification of eight coupling products. These coupling products were identified using ^1^H and ^13^C 1D NMR and HMBC and HSQC 2D NMR. The NMR spectral data and assignments are shown in Table 1, the key HMBC correlations are in Figure 3, and the detailed spectra with a description of the data analysis are in the Figures S6–S13. Among these eight coupling products, five different linkage types were found, namely, 4-*O*-7′/3-8′, 2-7′/8-8′, 8-8′/9-*N*-7′, 8-8′, and 4-*O*-8′. Since the large-scale oxidative coupling was at a less advanced stage of the reaction, an additional product (compound 27) could be purified and identified, even though compound 27 was a very minor product after two hours of small-scale oxidative coupling.

**Table 1:** ^1^H (600 MHz) and ^13^C (150 MHz) NMR Spectral Data for Hydroxycinnamoylagmatine Dimers in Methanol-*d*_4_; Assignments Were Based on HMBC and HSQC 2D Spectra

	product							
	CouAgm-4-*O*-7′/3-8′-DCouAgm (9)		FerAgm-8-8′-FerAgm (12)^a^		FerAgm-2-7′/8-8′-DFerAgm (13)		FerAgm-4-*O*-7′/3-8′-DFerAgm (18)	
position	δ_C_	δ_H_, mult. (J)^b^	δ_C_	δ_H_, mult. (J)	δ_C_	δ_H_, mult. (J)	δ_C_	δ_H_, mult. (J)
1	128.16		127.03		123.71		129.10	
2	124.20	7.38 (1H, s)	112.08	7.25 (2H, d, 1.74)	131.39		111.58	7.19 (1H, s)
3	127.44		147.72		115.54	6.45 (1H, s)	145.00	
4	161.30		148.12		148.41		150.20	
5	109.78	6.91 (1H, d, 8.50)	115.15	6.82 (2H, d, 8.29)	146.75		144.86	
6	129.04	7.53 (1H, d, 8.65)	124.53	7.10 (2H, dd, 8.39, 1.74)	111.72	6.96 (1H, s)	116.98	7.00 (1H, s)
7	140.10	7.50 (1H, d, 15.39)	140.78	7.90 (2H, s)	132.89	7.30 (1H, s)	140.42	7.48 (1H, d, 15.71)
8	117.70	6.48 (1H, d, 15.39)	126.66		127.52		118.12	6.49 (1H, d, 15.38)
9	167.80		166.79		169.63		167.77	
11	38.50	3.37 (4H, m)^c^	38.44	3.44 (4H, m)	38.60	3.29 (2H, m)	38.45	3.37 (2H, m)
12	25.76	1.66 (4H, m)	25.79	1.61 (6H, m)	25.80	1.31 (2H, m)	25.78	1.66 (4H, m)
13	26.33	1.66 (4H, m)	25.79	1.61 (6H, m)	25.80	1.31 (2H, m)	25.78	1.66 (4H, m)
14	40.75	3.24 (4H, m)^d^	40.73	3.21 (4H, m)	40.70	3.21 (2H, m)	40.60	3.24 (2H, m)
16	157.70		157.19		157.27		157.28	
MeO-C3			54.94	3.80 (6H, s)	55.32	3.90 (3H, s)	55.47	3.94 (3H, s)
MeO-C5								
1′	130.80				133.75		131.20	
2′	127.06	7.20 (2H, d, 8.50)			120.66	6.53 (1H, dd, 7.88, 1.64)	109.10	6.96 (1H, s)
3′	115.23	6.82 (2H, d, 8.94)			147.72		147.96	
4′	157.32				145.04		146.88	
5′	115.23	6.82 (2H, d, 8.94)			114.72	6.71 (1H, d, 8.18)	115.04	6.83 (2H, s)
6′	127.06	7.20 (2H, d, 8.50)			111.72	6.78 (1H, d, 1.74)	118.60	6.83 (2H, s)
7′	88.10	5.93 (1H, d, 7.77)			47.06	4.34 (1H, d, 6.96)	88.80	6.97 (1H, d, 8.35)
8′	56.60	4.20 1H, d, 7.58)			50.37	3.78 (1H, d, 6.85)	57.60	4.24 (1H, d, 8.02)
9′	172.10				173.93		171.80	
11′	38.50	3.36 (4H, m)^c^			38.35	3.06 (2H, m)	38.60	3.34 (2H, m)
12′	25.76	1.63 (4H, m)			25.80	1.31 (2H, m)	26.44	1.63 (4H, m)
13′	26.33	1.63 (4H, m)			25.80	1.31 (2H, m)	26.44	1.63 (4H, m)
14′	40.75	3.23 (4H, m)^c^			40.73	2.97 (2H, m)	40.68	3.23 (2H, m)
16′	157.70				157.27		157.28	
MeO-C3′					54.96	3.81 (3H, s)	55.06	3.85 (3H, s)
MeO-C5′								

	product							
	FerAgm-4-*O*-8′-FerAgm (21)		SinAgm-2-7′/8–8′-SinAgm (25)		SinAgm-8-8′-SinAgm (27)^a^		SinAgm-8-8′/9-*N*-7′-DSinAgm (29)	
position	δ_C_	δ_H_, mult. (J)	δ_C_	δ_H_, mult. (J)	δ_C_	δ_H_, mult. (J)	δ_C_	δ_H_, mult. (J)
1	130.6		n.d.		126.06		125.29	
2	111.07	7.32 (1H, d, 1.49)	123.36		107.23	6.96 (4H, s)	107.49	6.77 (2H, s)
3	149.23		145.4		147.95		148.01	
4	146.45		141.47		137.35		136.03	
5	113.7	6.81 (1H, d, 8.44)	147.84		147.95		148.01	
6	121.07	7.05 (1H, dd, 8.51, 1.62)	108.06	7.03 (1H, s)	107.23	6.96 (4H, s)	107.49	6.77 (2H, s)
7	139.78	7.47 (1H, d, 15.94)	134.18	7.57 (1H, s)	140.98	7.92 (2H, s)	134.99	7.53 (1H, d, 2.40)
8	119.6	5.63 (1H, d, 15.87)	125.7		127.43		126.2	
9	167.9		168.91		166.7		169.75	
11	38.52	3.37 (3H, m)^d^	38.71	3.30 (4H, m)^d^	38.7	3.45 (2H, m)	38.69	3.24 (2H, m)
12	25.67	1.56 (2H, m)	25.87	1.34 (2H, m)	25.35	1.64 (2H, m)	25.6	1.57 (4H, m)
13	26.35	1.65 (4H, m)	25.93	1.64 (2H, m)	26.4	1.21 (4H, m)	36.34	1.37 (4H, m)
14	40.68	3.24 (2H, m)	40.66	3.19 (5H, m)	40.8	2.95 (4H, m)	40.63	3.20 (4H, m)
16	157.25		157.38		157.25		157.23	
MeO-C3	55.18	4.03 (3H, s)	74.6	3.58 (3H, s)	55.4	3.83 (12H, s)	55.71	3.87 (6H, s)
MeO-C5			70.5	3.94 (3H, s)	55.4	3.83 (12H, s)	55.71	3.87 (6H, s)
1′	124.05		133.67				130.1	
2′	112.4	7.33 (1H, d, 1.69)	104.72	6.41 (2H, s)			103.67	6.57 (2H, s)
3′	147.55		147.56				148.53	
4′	148.08		n.d.				137.4	
5′	114.08	6.75 (1H, d, 8.17)	147.56				148.53	
6′	124.9	7.07 (1H, dd, 8.24, 1.76)	104.72	6.41 (2H, s)			103.67	6.57 (2H, s)
7′	123.89	7.27 (1H, s)	40.52	4.87 (2H, s)^d^			65.67	4.64 (n.d., d, 3.26)^d^
8′	140.5		48.68	3.85 (1H, d, 1.40)			53.28	4.00 (1H, m)
9′	164.62		173.13				171.8	
11′	38.52	3.35 (n.d., m)^d^	38.21	3.07 (2H, m)			38.8	3.13 (2H, m)
12′	25.67	1.53 (2H, m)	25.05	1.34 (2H, m)			25.6	1.57 (4H, m)
13′	26.35	1.65 (4H, m)	25.93	1.64 (2H, m)			36.34	1.37 (4H, m)
14′	40.68	3.17 (2H, m)	40.48	3.02 (2H, m)			40.64	3.08 (2H, m)
16′	157.25		157.38				157.23	
MeO-C3′	54.74	3.71 (3H, s)	70.35	3.73 (6H, s)			55.53	3.85 (6H, s)
MeO-C5′			70.35	3.73 (6H, s)			55.53	3.85 (6H, s)

a Prime positions are identical to the non-prime positions since the compound is symmetrical.

b s: singlet, d: doublet, t: triplet; dd: doublet of doublets, dt: doublet of triplets, and m: multiplet.

c These peaks overlap; therefore, the number of protons is the total number for both positions.

d Overlap with solvent peak; n.d. not defined.

### 3.4 Mass Spectrometric Characterization of Additional Coupling Products

**Figure 4:**
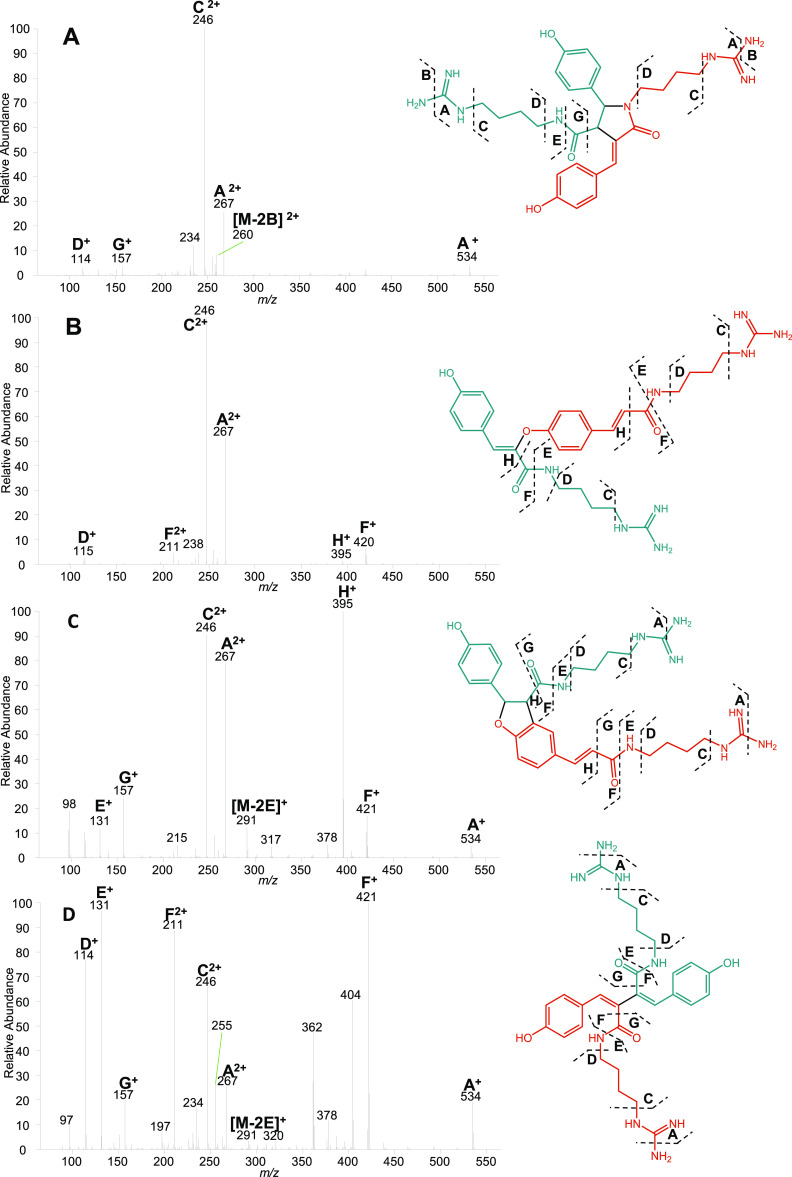
Four examples of ESI-IT-MS^2^ CID fragmentation spectra for different CouAgm dimers, namely, CouAgm-8-8′/9-N-7′-DCouAgm (A), CouAgm-4-O-8′-CouAgm (B), CouAgm-4-O-7′/3–8′-DCouAgm (C), and CouAgm-8-8′-DCouAgm (D). Peak labels show the corresponding fragmentation pathway as pictured in the structure and its measured *m*/*z*. Fragments were identified based on the elemental composition determined by ESI-FT-MS^2^ (Figure S14). Fragments that could not be identified based on the elemental composition as determined by ESI-FT-MS^2^ are unlabeled.

Based on the mass spectrometric fragmentation behavior of the purified compounds, other oxidative coupling products present in the reaction mixtures could also be tentatively identified. The molecular formula of the main fragments formed was identified via HRMS. Using the MS fragmentation spectra for all the dimeric products that were identified by NMR, six additional coupling products were tentatively identified. MS fragmentation spectra of all (tentatively) identified and unidentified compounds are given in Table S3. Since fragments for all coupling products are mostly related to the agmatine moiety (exemplified for CouAgm in Figure 4), it is not possible to distinguish different linkage types based on unique diagnostic fragments. Differences in the relative abundances of specific fragments, however, can be used for the tentative identification of the different linkage types. The similarities between the fragmentation spectra are clearly illustrated by comparison of the fragmentation spectra for 8-8′/9-*N*-7′-linked and 4-*O*-8′-linked dimers of the different precursors. These two linkage types could only be distinguished based on the [M + H – NH_2_]^+^ fragment (A^+^ in Figure 4), which had a relative abundance of ≥2% for 8-8′/9-*N*-7′-linked dimers, whereas this fragment had a relative abundance of <0.5% for the 4-*O*-8′-linked dimers. This is the only consistently observed difference between the fragmentation spectra of 8-8′/9-*N*-7′-linked and 4-*O*-8′-linked dimers formed from all three different precursors. The fragments formed for 2-7′/8-8′-linked and 8-8′-linked dimers were almost identical. Typical for the fragmentation spectra for the 8-8′-linked dimers was that the relative abundance for both the [M + H – M_agmatine_]^+^ and [M + 2H – M_agmatine_]^2+^ fragments (F^+^ and F^2+^ in Figure 4) was higher than 70%. Based on these criteria, we tentatively identified three dimers of CouAgm and one dimer of FerAgm. The fragmentation pattern for the 4-*O*-7′/3-8′-linked dimer could be recognized more easily since this was the only type of coupling product where the [M + H – CO – M_agmatine_]^+^ fragment (H^+^ in Figure 4) was the most abundant. Two isomers of the 4-*O*-7′/3-8′-linked dimer were identified for both CouAgm and FerAgm. These isomers could be tentatively identified since their fragmentation patterns were identical to those of the purified isomer that was identified by high-resolution MS and NMR. An overview of the mass spectrometric data of the (tentatively) identified products is given in Table 2. The full RP-UHPLC-MS data of all peaks and the ESI-IT-MS^2^ CID spectra of all (tentatively) identified dimers are shown in Tables S1 and S2, respectively.

**Table 2:** Spectral Data and (Tentative) Identifications of Hydroxycinnamoylagmatine Oxidative Coupling Products after 120 min of Incubation with HRP on a Small Scale^d^

	UHPLC-PDA-ESI-IT-MS					ESI-FT-MS				
compound	RT (min)	λ_max_ (nm)	ionization	*m*/*z*	MS^2^ (*m*/*z*) (relative abundance)^a^	molecular formula	calculated *m*/*z*	observed *m*/*z*	error (ppm)	annotation
CouAgm										
2	6.27	n.d.	[M + H]^+^	277	260 (100), 235 (41), 114 (24), 217 (20)	C_14_H_20_O_2_N_4_	277.16590	277.16565	–0.91	*cis*-CouAgm^b^
3	6.74	318	[M + 2H]^2+^	276	421 (100), 131 (90), 234 (79), 211 (76), 114 (72), 404 (53), 246 (53), 362 (46), 230 (30), 361 (28), 422 (26), 255 (23), 267 (20), 157 (20), 534 (18), 405 (15), 231 (15)	C_28_H_38_O_4_N_8_	276.15808	276.15771	–1.33	CouAgm-8-8′-DCouAgm^b^
5	9.55	294	[M + H]^+^	277	260 (100), 114 (17), 217 (17), 115 (12), 235 (10)	C_14_H_20_O_2_N_4_	277.16590	277.16568	–0.81	*trans*-CouAgm^c^
6	11.09	318	[M + 2H]^2+^	276	246 (100), 267 (29), 234 (11), 260 (5), 534 (3), 157 (3), 421 (2), 211 (2), 114 (2), 131 (2)	C_28_H_38_O_4_N_8_	276.15808	276.15793	0.53	CouAgm-8-8′/9-*N*-7′-DCouAgm^b^
9	19.69	310	[M + 2H]^2+^	276	395 (100), 246 (90), 267 (79), 157 (24), 396 (24), 98 (19), 421 (17), 291 (12), 131 (12), 114 (12), 97 (11), 420 (11), 534 (5), 211 (3)	C_28_H_38_O_4_N_8_	276.15808	276.15814	0.23	CouAgm-4-*O*-7′/3-8′-DCouAgm^c^
10	19.97	318	[M + 2H]^2+^	276	395 (100), 246 (100), 267 (91), 157 (24), 396 (24), 98 (17), 421 (17), 97 (13), 291 (12), 255 (12), 131 (10), 114 (9), 534 (4)	C_28_H_38_O_4_N_8_	276.15808	276.15808	–0.01	CouAgm-4-*O*-7′/3-8′-DCouAgm^b^
11	20.50	250	[M + 2H]^2+^	276	246 (100), 267 (51), 211 (4), 421 (4), 395 (1), 114 (1)	C_28_H_38_O_4_N_8_	276.15808	276.15802	–0.21	CouAgm-4-*O*-8′-CouAgm^b^
FerAgm										
12	10.25	334	[M + 2H]^2+^	306	131 (100), 241 (99), 481 (78), 114 (61), 297 (41), 157 (39), 464 (36), 276 (31), 323 (31), 422 (29), 227 (27), 482 (26), 285 (26), 179 (25), 298 (22), 421 (17), 594 (16), 436 (14), 465 (11), 351 (10)	C_30_H_42_O_6_N_8_	306.16864	306.16864	–0.20	FerAgm-8-8′-FerAgm^c^
13	10.38	334	[M + 2H]^2+^	306	131 (100), 481 (60), 464 (58), 241 (42), 114 (39), 297 (37), 157 (29), 227 (25), 422 (23), 323 (22), 436 (21), 594 (17), 285 (17), 465 (17), 340 (16), 482 (16), 298 (16), 276 (12), 439 (12), 331 (10)	C_30_H_42_O_6_N_8_	306.16864	306.16840	–0.79	FerAgm-2-7′/8–8′-DFerAgm^c^
14	12.25	318	[M + H]^+^	307	290 (100), 177 (22), 114 (18), 247 (14)	C_15_H_22_O_3_N_4_	307.17647	307.17633	–0.45	*trans*-FerAgm^c^
16	14.07	n.d.	[M + 2H]^2+^	306	276 (100), 297 (32), 285 (11), 131 (10), 157 (7), 481 (6), 241 (6), 114 (5), 594 (4), 290 (4)	C_30_H_42_O_6_N_8_	306.16864	306.16861	–0.10	FerAgm-8-8′/9-*N*-7′-DFerAgm^b^
18	21.06	322	[M + 2H]^2+^	306	455 (100), 297 (55), 276 (49), 157 (44), 481 (27), 456 (27), 131 (16), 351 (12), 114 (10)	C_30_H_42_O_6_N_8_	306.16864	306.16870	0.19	FerAgm-4-*O*-7′/3-8′-DFerAgm^c^
20	22.01	322	[M + 2H]^2+^	306	455 (100), 297 (53), 276 (51), 157 (44), 456 (25), 481 (24), 131 (15), 351 (9), 114 (8)	C_30_H_42_O_6_N_8_	306.16864	306.16855	–0.30	FerAgm-4-*O*-7′/3-8′-DFerAgm^b^
21	23.26	326	[M + 2H]^2+^	306	276 (100), 297 (48), 289 (14), 481 (4), 241 (2), 455 (2), 114 (1), 290 (1)	C_30_H_42_O_6_N_8_	306.16864	306.16855	–0.30	FerAgm-4-*O*-8′-FerAgm^c^
SinAgm										
25	8.87	330	[M + 2H]^2+^	336	131 (100), 361 (99), 157 (64), 328 (60), 383 (57), 306 (57), 327 (56), 370 (54), 271 (52), 541 (45), 315 (43), 114 (35), 344 (29), 301 (21), 496 (19), 362 (18), 181 (17), 654 (15), 524 (15), 542 (14)	C_32_H_46_O_8_N_8_	336.17921	336.17923	0.07	SinAgm-2-7′/8-8′-SinAgm^c^
27	11.21	338	[M + 2H]^2+^	336	271 (100), 131 (81), 315 (79), 306 (78), 541 (71), 411 (69), 383 (50), 157 (42), 328 (36), 327 (34), 114 (31), 542 (23) 524 (19), 344 (18), 361 (18), 412 (17), 370 (16), 387 (15), 231 (13)	C_32_H_46_O_8_N_8_	336.17921	336.17899	–0.65	SinAgm-8-8′-SinAgm^c^
29	13.72	330	[M + 2H]^2+^	336	306 (100), 327 (30), 315 (14), 157 (9) 131 (8), 320 (6), 114 (5), 271 (5), 541 (4), 654 (3)	C_32_H_46_O_8_N_8_	336.17921	336.17905	–0.47	SinAgm-8-8′/9-*N*-7′-DSinAgm^c^
30	14.18	322	[M + H]^+^	337	320 (100), 207 (55), 114 (18), 303 (14), 277 (12), 115 (10)	C_16_H_24_O_4_N_4_	337.18703	337.18674	–0.87	*trans*-SinAgm^c^

a All fragments with a relative abundance of 10% or higher and fragments linked to the fragmentation patterns (Figure 4) are reported.

b Identified based on the fragmentation pattern.

c Identified by NMR; n.d. = not detected.

d Compound numbers correspond to selected numbers from Figure 3. RP-UHPLC- MS data of all peaks are given in Table S1.

### 3.5 Formation of HCAgm Dimers with Five Different Linkage Types

Based on the assumption that all dimers had a similar UV_320_ response, we conclude that the major coupling products of CouAgm and FerAgm were 4-*O*-7′/3-8′-linked dimers (Table 3). This linkage type is also present in the main dimers described to naturally occur in barley.^1−3^ SinAgm did not form any coupling products that possessed this linkage type, which was expected since C-3 is already substituted with a methoxy group, hindering coupling at that position. Instead, the most abundant coupling product for SinAgm possessed a 2-7′/8-8′-linkage. CouAgm and FerAgm also formed coupling products with a 2-7′/8-8′-linkage, albeit at a lower abundance. The homodimer of FerAgm with this linkage type has previously been reported as murinamide A, a dimer naturally occurring in barley-related *Hordeum* species.^10^ In this study, the third linkage type known for HCAgm dimers in barley and related species, an 8-8′/9-*N*-7′-linkage, was also identified for the coupling of FerAgm and SinAgm. Ube et al.^10^ also showed that HRP can be used to form 4-*O*-7′/3-8′-linked, 2-7′/8-8′-linked, and 8-8′/9-*N*-7′-linked dimers of FerAgm. They reported that the 8-8′/9-*N*-7′-linked dimer was the most abundant product, which was a minor product in our case. However, they used alkaline conditions (9.3 mM NaOH, pH unknown), whereas we performed oxidative coupling at pH 7. No 8-8′/9-*N*-7′-linked dimer was formed when Ube et al.^10^ performed oxidative coupling under acidic conditions. Their results with FerAgm suggested that the reaction conditions may be used to modulate the product profile obtained upon oxidative coupling. We investigated this for oxidative coupling of CouAgm by performing the incubation at three different pH values (5, 7, and 8.5) but did not find major differences in the product profile at these three different pH values (Figure S15). It is unknown whether the effect of pH on oxidative coupling is substrate-dependent or whether this is due to different reaction conditions. Therefore, we conclude that more research on the effect of reaction conditions, such as pH and temperature, on the oxidative coupling of phenolamides by HRP is needed. Further understanding on the effect of the reaction conditions on the products formed would possibly allow us to steer the product profile, for example, to form coupling products with known bioactivities.

**Table 3:** Relative Amounts of the Different Linkage Types Formed per HCAgm after 120 min of Incubation with HRP on a Small Scale^a^

	relative amounts (± st. dev.)		
	CouAgm	FerAgm	SinAgm
4-*O*-7′/3-8′-linkage	62.4 (± 1.7)	57.9 (± 4.1)	0.0 (± 0.0)
2-7′/8-8′-linkage	0.0 (± 0.0)	18.3 (± 0.7)	60.6 (± 0.7)
8-8′/9-*N*-7′-linkage	7.3 (± 0.6)	2.0 (± 0.3)	25.4 (± 1.5)
4-*O*-8′-linkage	4.9 (± 0.0)	14.4 (± 0.4)	0.0 (± 0.0)
8-8′-linkage	14.0 (± 0.3)	4.8 (± 0.4)	14.0 (± 0.2)
unidentified	11.3 (± 0.6)	2.6 (± 0.5)	0.0 (± 0.0)

a Percentages are determined based on the relative UV_320_ peak area for all dimers. Standard deviations (st. dev.) are based on triplicates.

The two less prevalent linkage types that were identified in this work, 8-8′ and 4-*O*-8′, have not been previously reported for HCAgm dimers. On the other hand, these linkage types have been reported for lignanamides with a different amine group.^16^ The 8-8′-linked dimer of SinAgm was purified from the large-scale oxidative coupling product but was only a minor product at the more advanced state of the reaction that was observed in the small-scale reaction. This might indicate that this product is further converted during the reaction and could serve as a precursor for the more complex linkage types, such as the 2-7′/8-8′-linked and 8-8′/9-*N*-7′-linked dimers. A similar reaction pathway was proposed for the formation of dimeric compounds from avenanthramides, another class of phenolamides.^25^ The small-scale coupling reaction was monitored over time by analyzing intermediate time points by RP-UHPLC-PDA-IT-MS, showing that the peak area of the 8-8′-linked dimer increased in the first 60 min of the reaction but this peak had completely disappeared after 120 min. To determine whether the 8-8′-linked dimer is initially formed during the reaction and subsequently decreased when the reaction proceeds, the oxidative coupling was performed in an NMR tube inside a 700 MHz NMR while recording proton spectra every 1.15 min for 2 h. The integral of the unique H-7 proton signal for the 8-8′-linked dimer (δ_H_ 7.9 ppm) was used to quantitatively track this linkage type over time. However, compared to the other proton peaks in the spectra, the intensity of the H-7 signal was very low. Due to the low intensity, a reliable peak integration was not possible and resulted in inconclusive results. Nonetheless, we hypothesize that this dimer is either an intermediate that is later converted into other linkage types, presumably the 2-7′/8-8′-linked and 8-8′/9-*N*-7′-linked dimers, or the dimer is further converted into larger oligomers. Further mechanistic investigation of this oxidative coupling reaction was beyond the scope of this work.

### 3.6 Oligomerization of HCAgms upon Oxidative Coupling

HCAgm dimers are expected to be involved in subsequent oxidative coupling reactions that can result in the formation of oligomeric reaction products that cannot be detected with RP-UHPLC-MS.^26^ Therefore, the presence of oligomeric reaction products was investigated using MALDI-TOF-MS (Figure S16). For CouAgm, FerAgm, and SinAgm, products with a degree of polymerization (DP) of up to 6, 5, and 4, respectively, were detected. For another type of phenolamide, that is, feruloyltyramine, trimers were also reported.^27−29^ For SinAgm, oxidative coupling resulted in a relatively limited oligomer formation, which is in accordance with the literature in which incubation of sinapic acid with HRP also yielded products restricted to a low DP.^30^ A possible explanation for this could be the presence of two methoxy groups on the aromatic ring, which restricts the linkage types that can possibly be formed for SinAgm. Based on our results, CouAgm and FerAgm oligomers with DPs > 2 are readily formed via peroxidase-catalyzed oxidative coupling, which suggests that they may also occur naturally in stressed barley, even though this has not yet been described in the literature.

### 3.7 Biomimetics of HRP-Catalyzed Oxidative Coupling of HCAgms and Future Outlook

This study aimed to gain insight in hydroxycinnamoylagmatine (HCAgm) reactivity and the coupling products formed upon in vitro enzymatic oxidative coupling. We have demonstrated that oxidative coupling of HCAgms by HRP is a useful tool to produce the naturally occurring antifungal compounds from barley and related *Hordeum* species. The fact that in vitro oxidative coupling with HRP yields the naturally occurring dimers as the main products supports the theory that barley peroxidases could perform the oxidative coupling step in the biosynthesis of these compounds.^2^ To verify previous findings reporting that dimers in *Hordeum vulgare* solely possess the 4-*O*-7′/3-8′-linkage, a barley (*H. vulgare*) extract was screened for the presence of dimers with the various linkage types. Based on comparison of the data resulting from that screening with chromatographic and mass spectrometric data obtained for the purified compounds presented in this manuscript, we conclude that only dimers with a 4-*O*-7′/3-8′-linkage were detected (data not shown). This is in line with previous reports that this is the only linkage type in *Hordeum vulgare*. Furthermore, our biomimetic approach for in vitro production of these compounds facilitates investigation of the bioactivity of HCAgms and their coupling products in future studies. For various types of phenolamides and their dimeric coupling products, bioactivities are reported in the literature; however, the structure–bioactivity relationships are still poorly understood.^31^ Better understanding of the bioactivity of HCAgms and their coupling products will contribute to a better understanding of the possible health benefits of incorporating barley or barley-derived products into the human or animal diet. Furthermore, after establishing the scope of antifungal activity of the dimeric compounds obtained from barley, they may be used as natural preservatives for food or feed products.

## Supporting Information Available

Determination of the enzyme activity, thermal and light stability data for the three types of hydroxycinnamoylagmatine, and IT-MS data, FT-MS data, and 2D NMR spectra for all purified compounds, absorbance of ABTS in a HRP-H_2_O_2_ system measured at 405 nm in triplicate, peak areas of *cis* (orange) and *trans* isomers (white) of CouAgm, FerAgm, and SinAgm monomers as determined using RP-UHPLC-FT-MS in PI in samples incubated at different temperatures for 10 and 60 min in the absence of light or with exposure to light, peak areas of *cis* (orange) and *trans* isomers (white) of CouAgm, FerAgm, and SinAgm monomers as determined using RP-UHPLC-FT-MS in PI in samples exposed to daylight for several days at room temperature, RP-UHPLC-PDA-IT-MS base peak chromatograms (*m*/*z* 250–1500) in the positive ionization mode for the large scale oxidative coupling of CouAgm, FerAgm, and SinAgm after 120 min, RP-UHPLC-PDA-IT-MS positive ionization mode base peak chromatograms of the coupling products of CouAgm, FerAgm, and SinAgm after 120 min, ^1^H (600 MHz) and ^13^C (150 MHz) NMR spectra and HMBC and HSQC correlations for the identified coupling products, UHPLC-PDA-ESI-IT-MS data and ESI-FT-MS data for the hydroxycinnamoylagmatine oxidative coupling products after 120 min incubation with HRP, ESI-IT-MS^2^ CID spectra for the different linkage types per monomer, identification of the main fragments in ESI-IT-MS^2^ CID spectra by determination of the elemental composition of the fragments by ESI-FT-MS^2^, RP-UHPLC-PDA-IT-MS base peak chromatograms (*m*/*z* 250–1500) in the positive mode after 120 min of incubation of CouAgm with HRP and H_2_O_2_ at pH 5, pH 7, and pH 8.5, and MALDI-TOF-MS spectra of CouAgm, FerAgm, and SinAgm incubated with HRP at *t* = 120 min (PDF)
