# Supplementary material for: Biomimetic Enzymatic Oxidative Coupling of Barley Phenolamides: Hydroxycinnamoylagmatines
Source: J Agric Food Chem. 2022 Dec 14;70(51):16241–52. doi: 10.1021/acs.jafc.2c07457 (PMC9801423; doi:10.1021/acs.jafc.2c07457)
Supplement: Supplementary file 1 — jf2c07457_si_001.pdf [file jf2c07457_si_001.pdf]

Biomimetic enzymatic oxidative coupling of barley phenolamides: hydroxycinnamoylagmatines.

Annemiek van Zadelhoff, Lieke Meijvogel, Anna-Marie Seelen, Wouter J.C. de Bruijn, Jean-Paul Vincken

Supporting information

HRP activity

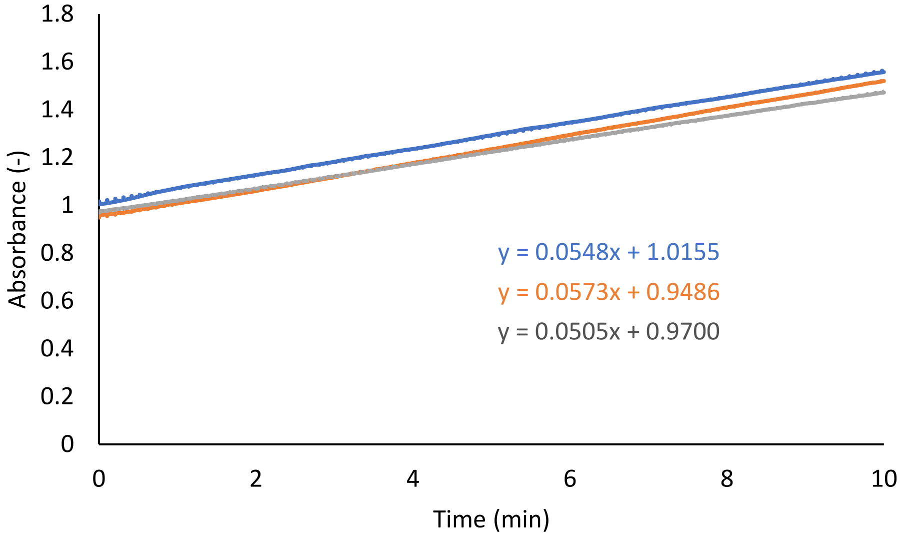

Figure S1. Absorbance of ABTS in a HRP-H_2O_2 system measured at 405 nm in triplicate. The enzyme activity was determined based on the average of these three measurements.

Temperature stability hydroxycinnamoylagmatines

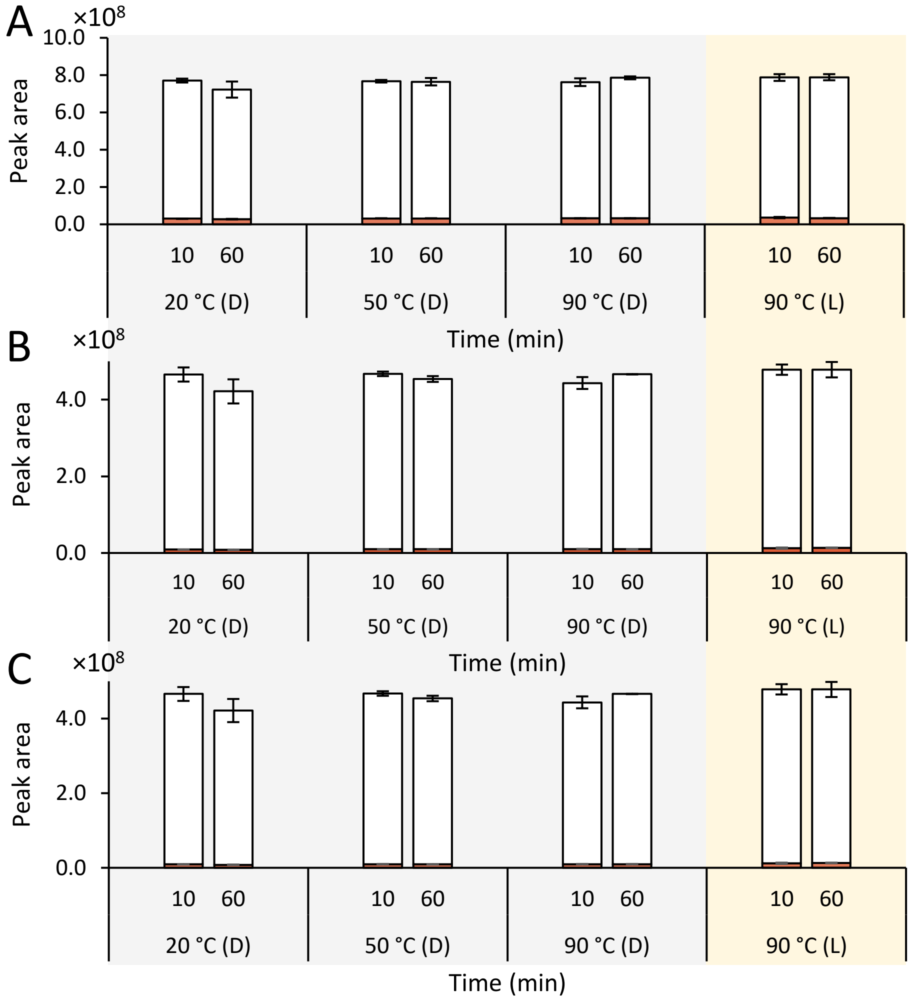

Figure S2. The peak areas of cis- (orange) and trans isomers (white) of CouAgm (A), FerAgm (B), and SinAgm (C) monomers as determined using RP-UHPLC-FT-MS in PI, in samples incubated at different temperatures for 10 and 60 minutes in absence of light (D) or with exposure to light (L). Error bars represent the standard deviation based on duplicates.

Light stability hydroxycinnamoylagmatines

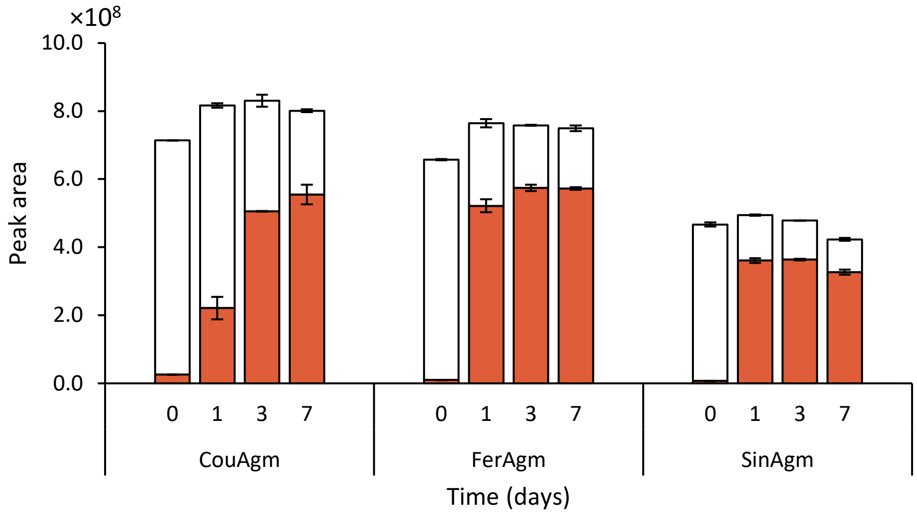

Figure S3. The peak areas of cis- (orange) and trans isomers (white) of CouAgm, FerAgm and SinAgm monomers as determined using RP-UHPLC-FT-MS in PI, in samples exposed to daylight for several days at room temperature. Error bars represent the standard deviation based on duplicates.

Large scale oxidative coupling

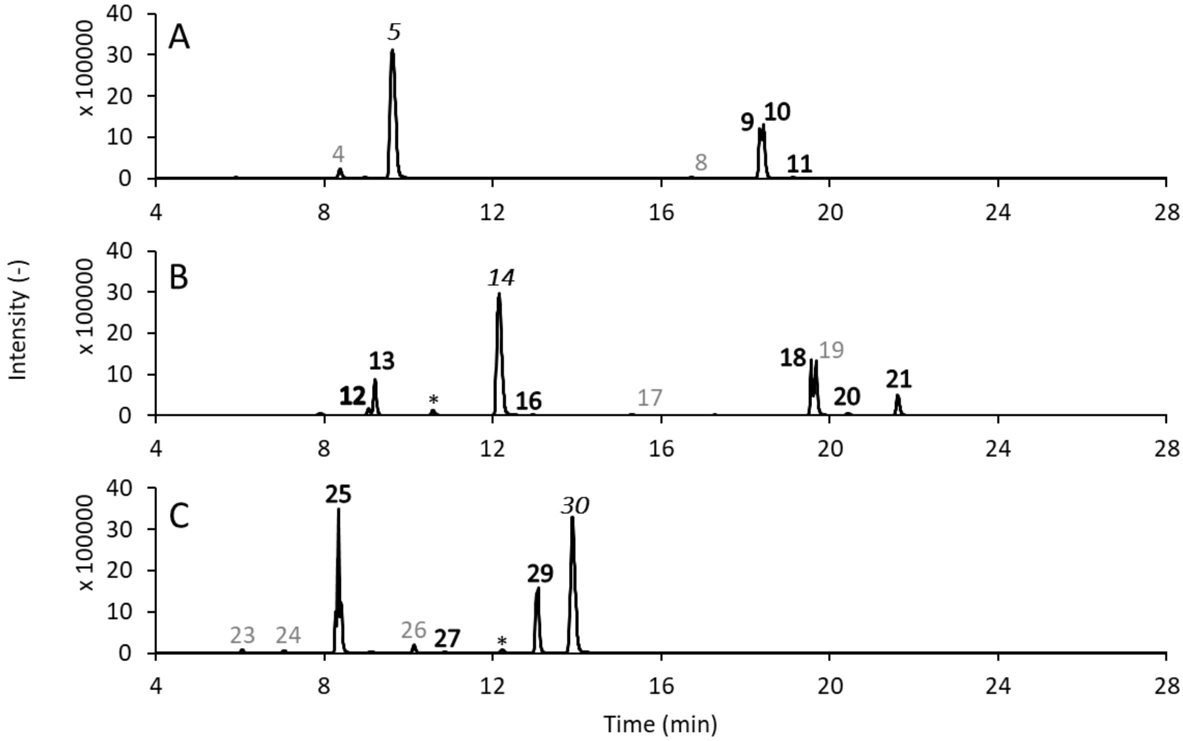

*Figure S4. RP-UHPLC-PDA-IT-MS base peak chromatograms (m/z 250-1500) in positive ionisation mode for the large scale oxidative coupling of CouAgm (A), FerAgm (B), and SinAgm (C) after 120 minutes. Peak numbers indicate monomers (black, italics), identified coupling products (black, bold), and unidentified compounds (grey). * Indicates an impurity present since the beginning of the reaction, which was not converted during the oxidative coupling reaction. The large scale and small scale oxidative coupling were analysed separately, which resulted in a retention time shift. Compounds present were compared to the small scale oxidative coupling based on MS^2.*

Purification of flash fractions

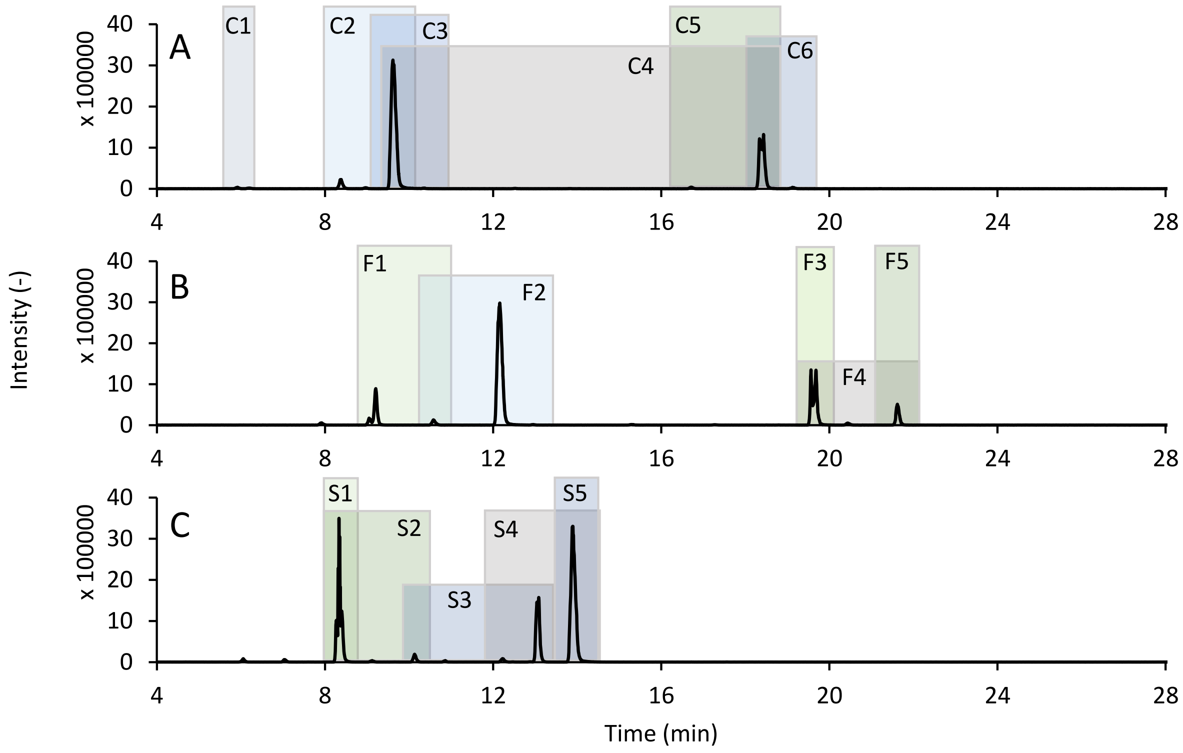

Figure S5. RP-UHPLC-PDA-IT-MS positive ionisation mode base peak chromatograms of the coupling products of CouAgm (A), FerAgm (B), and SinAgm (C) after 120 minutes. Pools obtained after separation using flash chromatography are indicated with the coloured boxes. Green pools were selected and further purified by preparative chromatography.

The selected fractions were most suitable for further purification, as these pools contained only two or three main compounds and contained enough material. Pools that were not selected either contained a too small amount of material or were mainly composed of the monomeric precursor. The following gradients were used for preparative chromatography of the selected pools.

Fraction C5: Isocratic at 10 % B for 1.20 min, 1.20-50.20 min linear gradient to 17 % B, 50.20-53.70 min linear gradient to 95 % B, 53.70-71.20 min isocratic at 95 % B.
Fraction F1: Isocratic at 5 % B for 1.20 min, 1.20-50.20 min linear gradient to 12 % B, 50.20-53.70 min linear gradient to 95 % B, 53.70-71.20 min isocratic at 95 % B.
Fraction F3: Isocratic at 10 % B for 1.20 min, 1.20-50.20 min linear gradient to 17 % B, 50.20-53.70 min linear gradient to 95 % B, 53.70-71.20 min isocratic at 95 % B.
Fraction F5: Isocratic at 10 % B for 1.20 min, 1.20-50.20 min linear gradient to 17 % B, 50.20-53.70 min linear gradient to 95 % B, 53.70-71.20 min isocratic at 95 % B.
Fraction S2: Isocratic at 8 % B for 1.20 min, 1.20-50.20 min linear gradient to 15 % B, 50.20-53.70 min linear gradient to 95 % B, 53.70-71.20 min isocratic at 95 % B.

CouAgm-4-*O*-7′/3-8′-DCouAgm (9)

Compound 9 had the molecular formula $C_{28}H_{38}O_4N_8$ as determined by HRMS at *m/z* 276.15814 $[M+2H]^{2+}$ (calculated for $C_{28}H_{40}O_4N_8^{2+}$, 276.15808). The ^1H and ^{13}C NMR spectra (Main text; Table 2) revealed the presence of two agmatine moieties [δ_H 3.37 (4H, m), δ_H 1.66 (4H, m), δ_H 3.24 (4H, m) and δ_C 38.50, 25.76, 26.33, 40.75, 157.70; δ_H 3.36 (4H, m), δ_H 1.63 (4H, m), δ_H 3.23 (4H, m) and δ_C 38.50, 25.76, 26.33, 40.75, 157.70], one 1,3,4-trisubstituted benzene ring at [δ_H 7.38 (1H, s), δ_H 6.91 (1H, d, J = 8.50 Hz), δ_H 7.53 (1H, d, J = 8.65 Hz) and δ_C 128.16, 124.20, 127.44, 161.30, 109.78, 129.04], one symmetrical 1,4-disubstituted benzene ring [δ_H 7.20 (2H, d, J = 8.50 Hz), δ_H 6.82 (2H, d, J = 8.94 Hz) and δ_C 130.80, 127.06, 115.23, 157.32, 115.23, 127.06], a pair of *trans*-olefinic protons at [δ_H 6.48 (1H, d, J = 15.39 Hz), δ_H 7.50 (1H, d, J = 15.39 Hz) and δ_C 140.10, 117.70], one methine [δ_H 4.20 (1H, d, J = 7.58 Hz) and δ_C 56.60], one oxymethine [δ_H 5.93 (1H, d, J = 7.77 Hz) and δ_C 88.10], and two conjugated carbonyl carbons (δ_C 167.80, 172.10). Using the combined analysis of HSQC and HMBC data, the 2D structure of the compound was constructed.

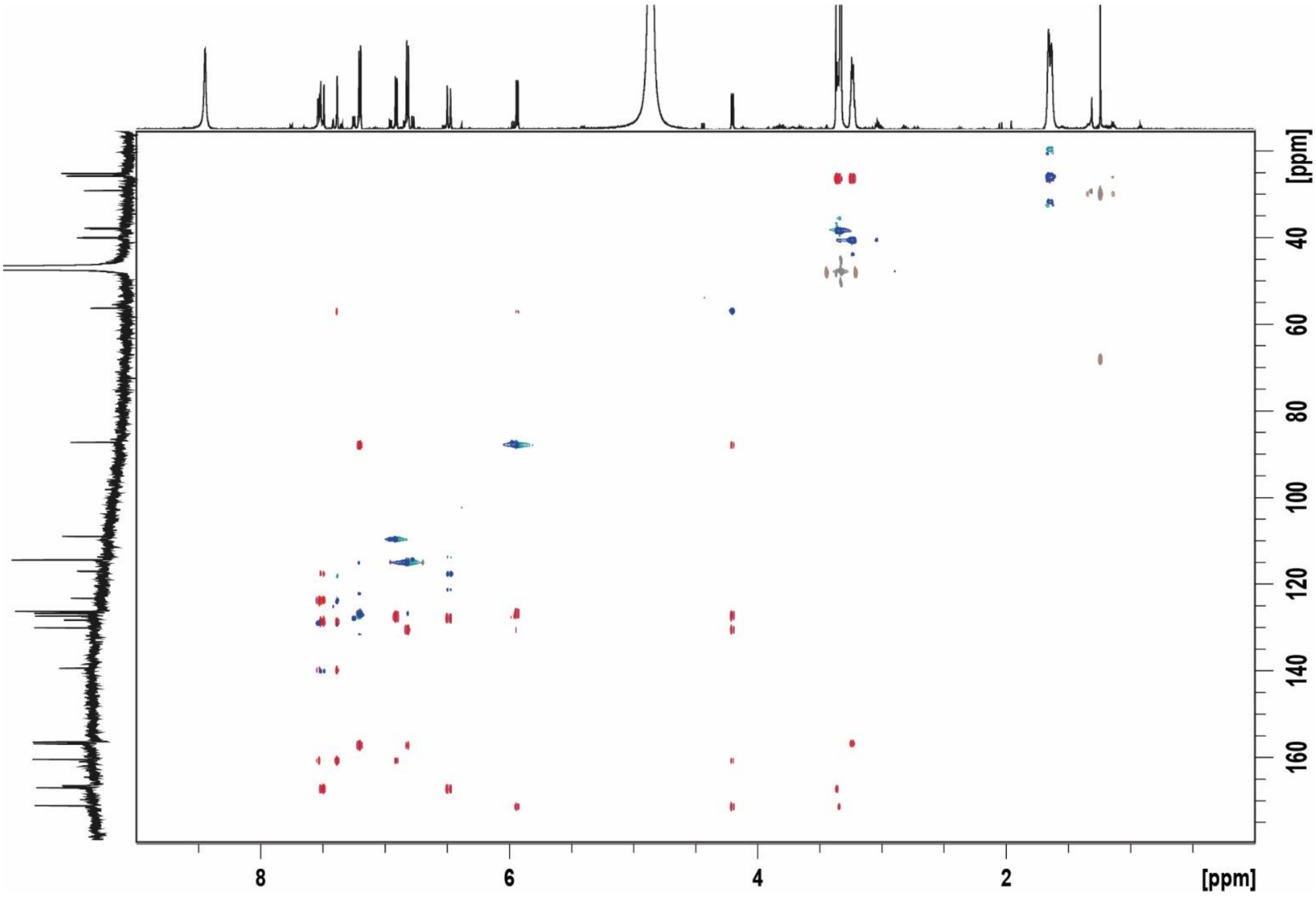

Figure S6. ^1H (600 MHz) and ^{13}C (150 MHz) NMR spectra and HMBC (blue/turquoise) and HSQC (red) correlations for CouAgm-4-O-7′/3-8′-DCouAgm (9). Impurities in the spectrum are shown in grey (HMBC) and brown (HSQC).

Compounds **(12)** and **(13)** were not separated by preparative chromatography and were thus measured as a mixture. NMR resonances could be assigned to either one of the compounds by using HSQC and HMBC data (Figure S8 and S9).

FerAgm-8-8′-FerAgm (12).

Compound 12 had the molecular formula $C_{30}H_{42}O_6N_8$ as determined by HRMS at *m/z* 306.16864 $[M+2H]^{2+}$ (calculated for $C_{30}H_{44}O_6N_8^{2+}$, 306.16864). The ^1H and ^{13}C NMR spectra (Main text; Table 2) of this dimer are similar to the ^1H and ^{13}C NMR spectra for the precursor of this dimer, feruloylagmatine.

Both the proton and carbon NMR spectrum for feruloylagmatine differ in two positions from the spectra obtained for this compound, being two (*trans*)-olefinic protons at [δ_H 7.42 (1H, d, J = 15.7 Hz), δ_H 6.56 (1H, d, J = 15.7 Hz) and for carbon δ_C 139.0, 119.0][1]. These signals are missing in this dimer, however one divergent proton and two divergent carbon signals are present in the spectra obtained for the dimer, being a singlet at [δ_H 7.90 (2H, s) linked to δ_C 140.78] and a resonance at δ_C 126.66. The loss of one of the proton signals of one of the protons linked to the carbon atoms present in the alkane group indicated that one of these carbons is involved in the linkage formed. Since no other positions showed a similar sign of substitution, it was concluded that the dimer formed is symmetrical. This indicates a symmetrical dimer was formed by an 8-8'-linkage, since HMBC showed a correlation between δ_C 140.78 and H-2 and H-6, proving that the proton resonance corresponds to H-7. This is comparable to the ^1H and ^{13}C NMR spectra for cannabisin G, an 8-8'-linked lignanamide present in *Cannabis sativa*[2].

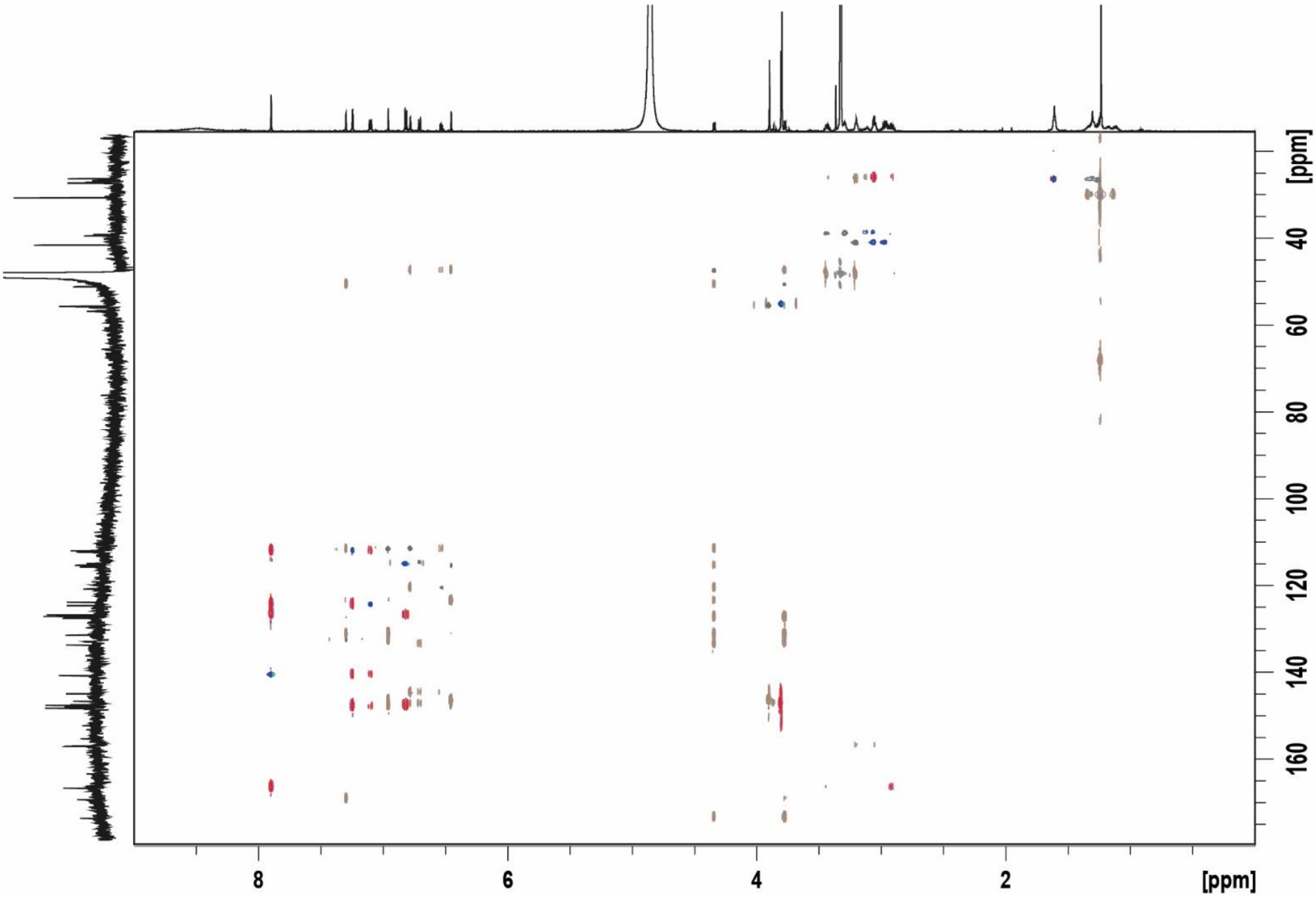

Figure S7. ^1H (600 MHz) and ^{13}C (150 MHz) NMR spectra and HMBC (blue/turquoise) and HSQC (red) correlations for FerAgm-8-8'-FerAgm (12). Impurities and correlations related to compound (13) in the spectrum are shown in grey (HMBC) and brown (HSQC). NMR data for compound 13 is given in Figure S8.

FerAgm-2-7′/8-8′-DFerAgm (13).

Compound 13 had the molecular formula $C_{30}H_{42}O_6N_8$ as determined by HRMS at *m/z* 306.16840 $[M+2H]^{2+}$ (calculated for $C_{30}H_{44}O_6N_8^{2+}$, 306.16864). The ^{1}H and ^{13}C NMR spectra (Main text; Table 2) match with a feruloylagmatine dimer reported by Ube et al.[3] as murinamide A. HSQC and HMBC correlations also confirmed the structure of this dimer.

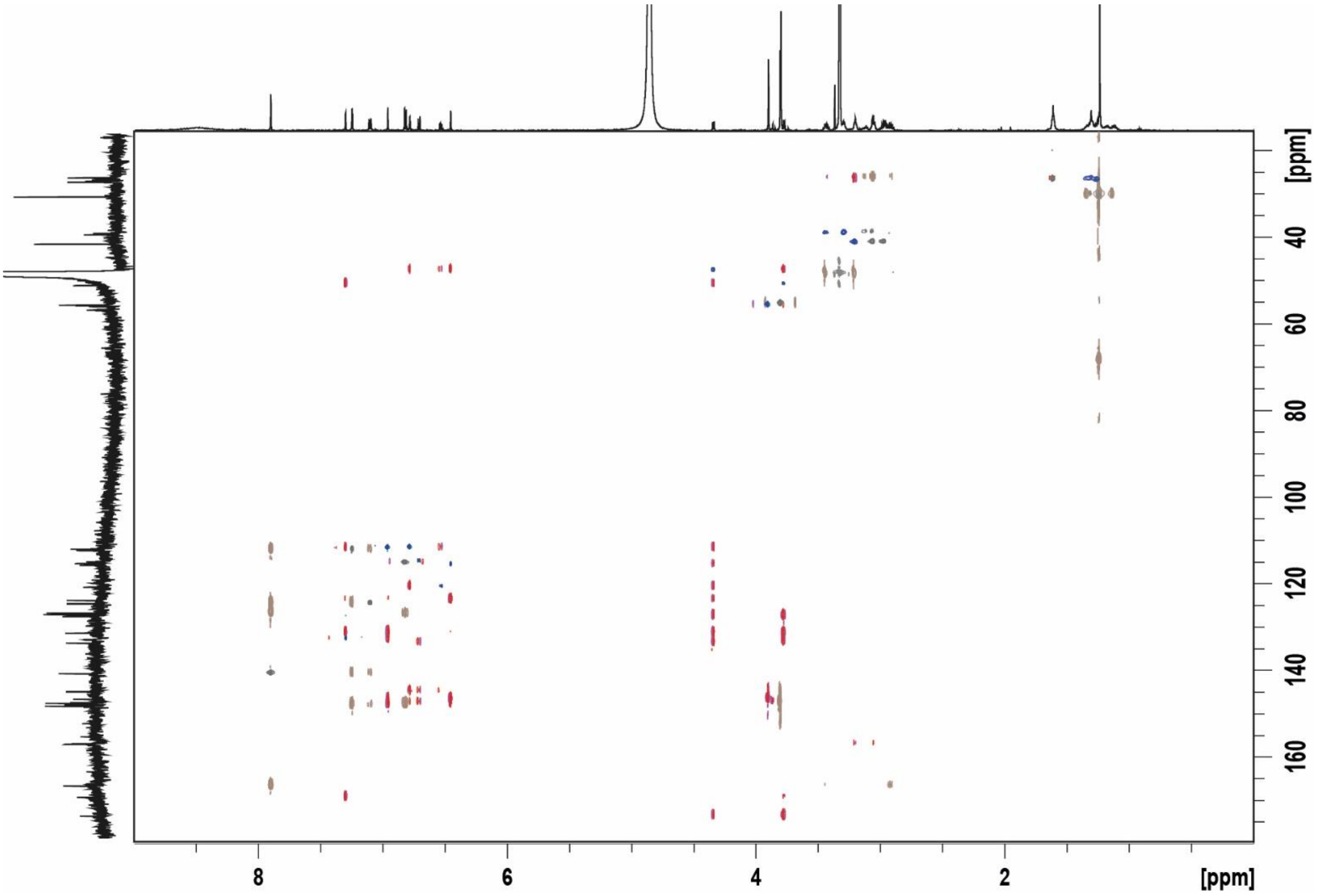

Figure S8. ^{1}H (600 MHz) and ^{13}C (150 MHz) NMR spectra and HMBC (blue/turquoise) and HSQC (red) correlations for FerAgm-2-7′/8-8′-DFerAgm (13). Impurities and correlations related to compound (12) in the spectrum are shown in grey (HMBC) and brown (HSQC). NMR data for compound 12 is given in Figure S7.

FerAgm-4-*O*-7′/3-8′-DFerAgm (18).

Compound 18 had the molecular formula $C_{30}H_{42}O_6N_8$ as determined by HRMS at *m/z* 306.16870 $[M+2H]^{2+}$ (calculated for $C_{30}H_{44}O_6N_8^{2+}$, 306.16864). The ^1H and ^{13}C NMR spectra (Main text; Table 2) revealed the presence of two agmatine moieties [δ_H 3.37 (4H, m), δ_H 1.66 (4H, m), δ_H 3.24 (4H, m) and δ_C 38.45, 25.78, 26.44, 40.60, 157.28; δ_H 3.34 (4H, m), δ_H 1.63 (4H, m), δ_H 3.23 (4H, m) and δ_C 38.60, 25.78, 26.44, 40.68, 157.28], one 1,3,4-trisubstituted benzene ring at [δ_H 6.96 (1H, s), δ_H 6.83 (2H, s), and δ_C 131.20, 109.10, 147.96, 146.88, 115.04, 118.60], one 1,3,4,5-tetraubstituted benzene ring [δ_H 7.19 (1H, s), δ_H 7.00 (1H, s) and δ_C 129.10, 111.58, 145.00, 150.20, 144.86, 116.98], a pair of (*trans*)-olefinic protons at [δ_H 6.49 (1H, d, J = 15.71 Hz), δ_H 6.49 (1H, d, J = 15.38 Hz) and δ_C 140.42, 118.12], one methine [δ_H 4.24 (1H, d, J = 8.02 Hz) and δ_C 57.60], one oxymethine [δ_H 6.97 (1H, d, J = 8.35 Hz) and δ_C 88.80], two methoxy groups [δ_H 3.94 (3H, s), δ_H 3.85 (3H, s) and δ_C 55.47, 55.06], and two conjugated carbonyl carbons (δ_C 167.77, 171.80). Based on the HMBC spectrum and coherence with previously reported NMR spectra the compound was assigned to be FerAgm-4-*O*-7′/3-8′-DFerAgm, also known as hordatine C.[3, 4]

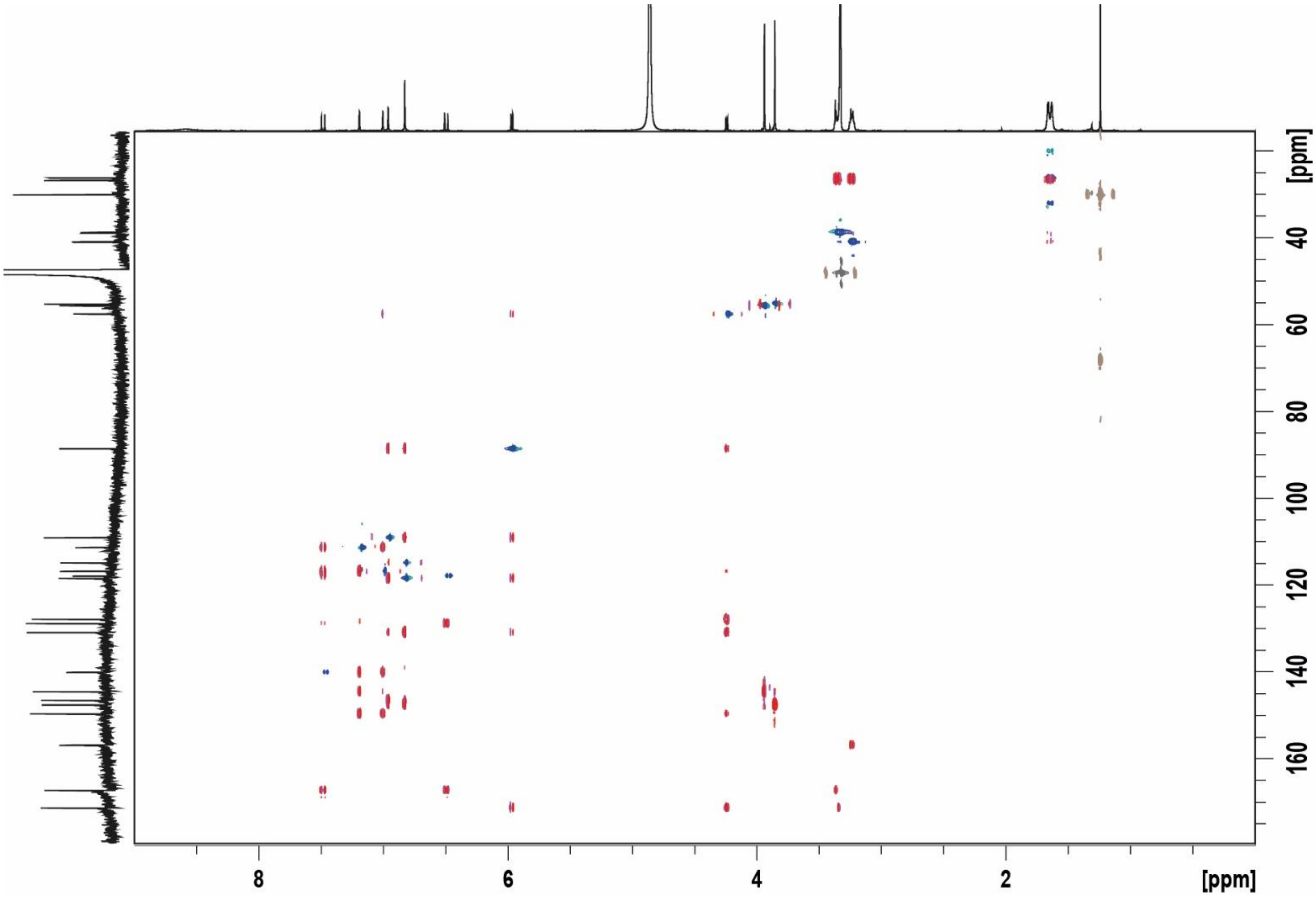

Figure S9. ^1H (600 MHz) and ^{13}C (150 MHz) NMR spectra and HMBC (blue/turquoise) and HSQC (red) correlations for FerAgm-4-O-7′/3-8′-DFerAgm (18). Impurities in the spectrum are shown in grey (HMBC) and brown (HSQC).

FerAgm-4-*O*-8′-FerAgm (21).

Compound 21 had the molecular formula $C_{30}H_{42}O_6N_8$ as determined by HRMS at *m/z* 306.16870 $[M+2H]^{2+}$ (calculated for $C_{30}H_{44}O_6N_8^{2+}$, 306.16855). The ^1H and ^{13}C NMR spectra (Main text; Table 2) revealed the presence of two agmatine moieties [δ_H 3.37 (3H, m), δ_H 1.53 (2H, m), δ_H 1.56 (2H, m), δ_H 1.65 (4H, m), δ_H 3.17 (2H, m), δ_H 3.24 (2H, m) and δ_C 38.52, 25.67, 26.35, 40.68, 157.25], two 1,3,4-trisubstituted benzene rings at [δ_H 7.32 (1H, d, J = 1.49 Hz), δ_H 6.81 (1H, d, J = 8.44 Hz), δ_H 7.05 (1H, dd, J = 8.51, 1.62 Hz), and δ_C 130.60, 111.07, 149.23, 146.45, 113.70, 121.07; δ_H 7.33 (1H, d, J = 1.69 Hz),

δ_H 6.75 (1H, d, J = 8.17 Hz), δ_H 7.07 (1H, dd, J = 8.24, 1.76 Hz), and δ_C 124.05, 112.40, 147.55, 148.08, 114.08, 124.90], a pair of (*trans*)-olefinic protons at [δ_H 7.47 (1H, d, J = 15.94 Hz), δ_H 5.63 (1H, d, J = 15.87 Hz) and and δ_C 139.78, 119.60], one methine [δ_H 7.27 (1H, s) and δ_C 123.89], two methoxy groups [δ_H 4.03 (3H, s), δ_H 3.71 (3H, s) and δ_C 55.18, 54.74], and two conjugated carbonyl carbons (δ_C 167.90, 164.62). The ^{1}H and ^{13}C NMR spectra were comparable to the spectra of cannabisin F, a 4-*O*-8′-linked lignanamide present in *Cannabis sativa*.[2]

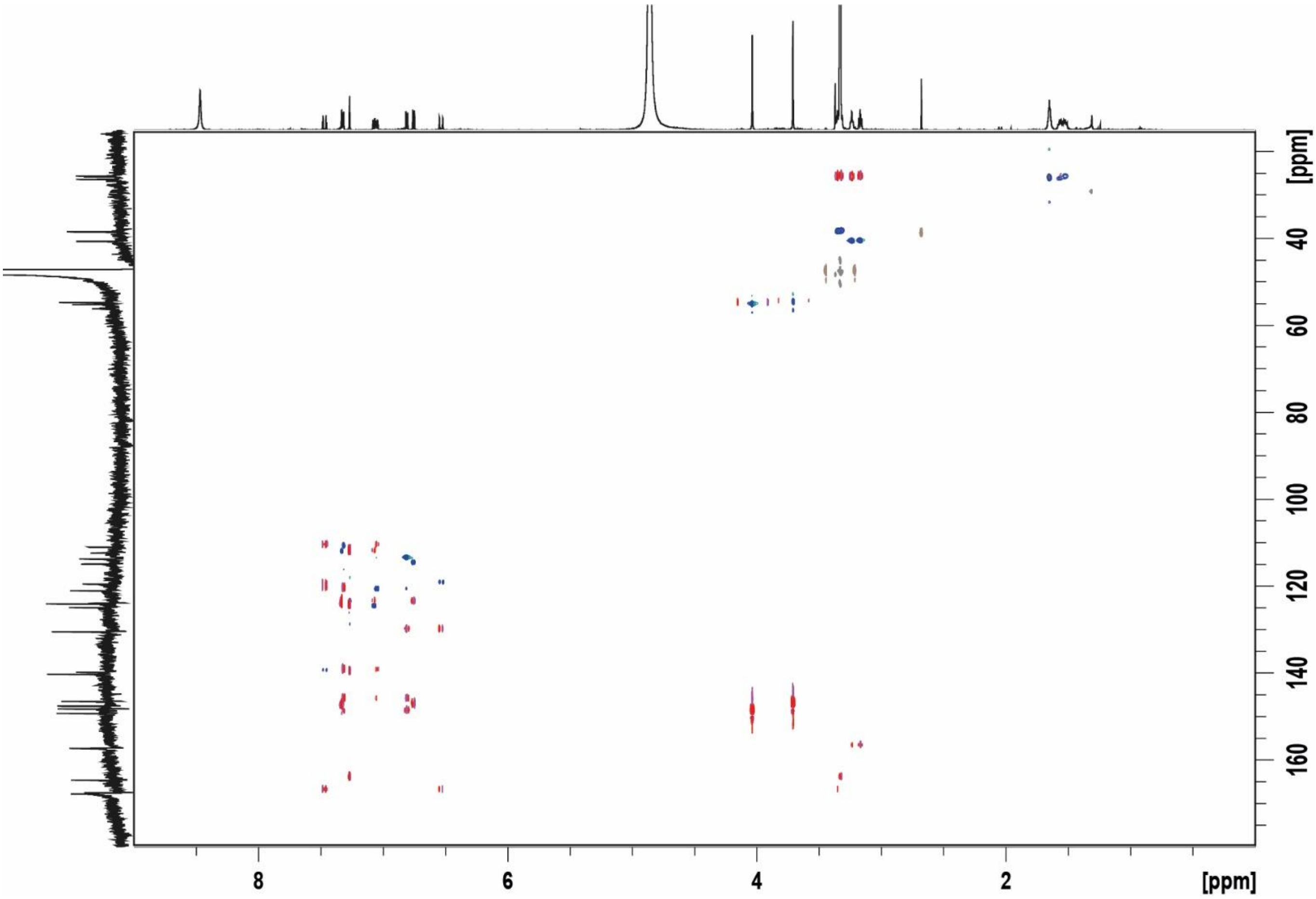

Figure S10. ^{1}H (600 MHz) and ^{13}C (150 MHz) NMR spectra and HMBC (blue/turquoise) and HSQC (red) correlations for FerAgm-4-O-8′-FerAgm (21). Impurities in the spectrum are shown in grey (HMBC) and brown (HSQC).

SinAgm-2-7′/8-8′-DSinAgm (25).

Compound 25 had the molecular formula $C_{32}H_{46}O_8N_8$ as determined by HRMS at *m/z* 336.17923 $[M+2H]^{2+}$ (calculated for $C_{32}H_{48}O_8N_8^{2+}$, 336.17921). The ^{1}H and ^{13}C NMR spectra (Main text; Table 2) revealed the presence of two agmatine moieties [δ_H 3.30 (4H, m), δ_H 1.34 (2H, m), δ_H 1.64 (2H, m), δ_H 3.19 (5H, m) and δ_C 38.71, 25.87, 25.93, 40.66, 157.38; δ_H 3.07 (2H, m), δ_H 1.34 (2H, m), δ_H 1.64 (2H, m), δ_H 3.02 (2H, m) and δ_C 38.21, 25.05, 25.93, 40.48, 157.38], one 1,3,4,5-tetrasubstituted benzene ring at [δ_H 6.41 (2H, s), and δ_C 133.67, 104.72, 147.56], one 1,3,4,5,6-pentasubstituted benzene ring at [δ_H 7.03 (1H, s), and δ_C 123.36, 145.40, 141, 47, 147.84, 108.06], one methine at [δ_H 7.57 (1H, s), and δ_C 134.18], four methoxy groups [δ_H 3.58 (3H, s), δ_H 3.94 (3H, s), δ_H 3.73 (6H, s), and δ_C 74.60, 70.50, 70.35], three carbon resonances at (δ_C 125.70, 40.52, 48.68), and two conjugated carbonyl carbons (δ_C 168.91, 173.13). Since three signals corresponding to methoxy groups were detected it was concluded that one of the two rings was no longer symmetrical and therefore must be involved in the linkage type formed. This was also indicated by the presence of a pentasubstituted ring. In the ^{1}H data both (*trans*)-olefinic proton pairs from the sinapoylagmatine precursor were no longer detected, and from the HMBC data it could be observed that C-2 correlates with both H-7′ and H-8′. Altogether, this showed that a ring shaped linkage was formed involving C-2, C-8, C-7′, and C-8′. This is the same linkage

as identified for compound 13, which is also reflected by the similarities in both the ^{1}H and ^{13}C NMR spectra. For compound 25 the signal obtained was lower, resulting in the lack of signal for C-1 and C-4′. However, based on the correlations in the HMBC data and the similarities with the data for compound 13, this linkage type could still be identified as a 2-7′/8-8′-linkage. This linkage type was also reported for a sinapoyltyramine homodimer called corydalisin C, which had ^{1}H and ^{13}C NMR spectra similar to the spectra obtained for compound 25.[5]

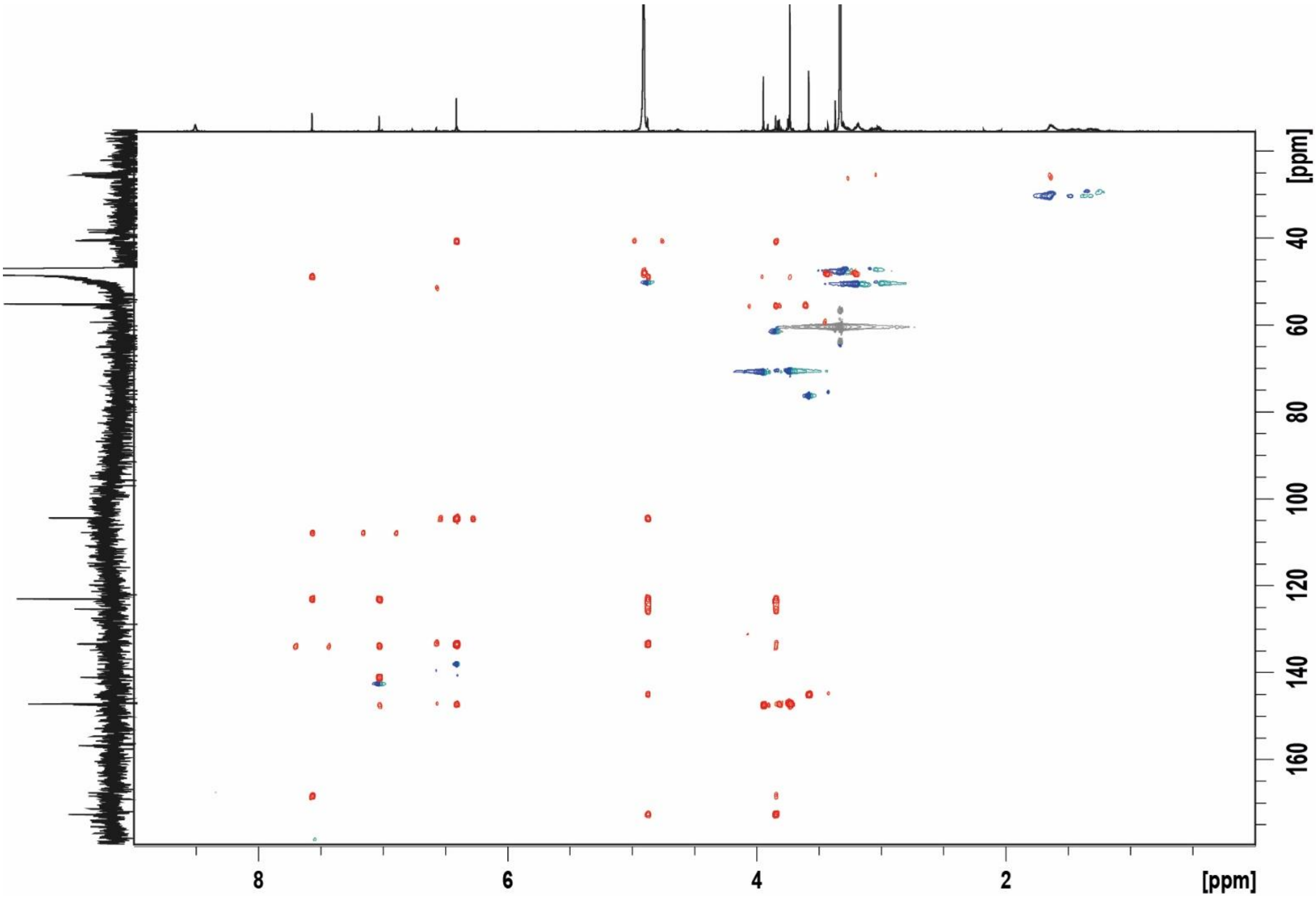

Figure S11. ^{1}H (600 MHz) and ^{13}C (150 MHz) NMR spectra and HMBC (blue/turquoise) and HSQC (red) correlations for SinAgm-2-7′/8-8′-SinAgm (25). Impurities in the spectrum are shown in grey (HMBC) and brown (HSQC).

SinAgm-8-8′-SinAgm (27).

Compound 27 had the molecular formula $C_{32}H_{46}O_8N_8$ as determined by HRMS at *m/z* 336.17899 $[M+2H]^{2+}$ (calculated for $C_{32}H_{48}O_8N_8^{2+}$, 336.17921). Similar as observed for FerAgm-8-8′-FerAgm (12), the ^1H and ^{13}C NMR spectra (Main text; Table 2) contained a limited number of resonances of which almost all resonances match with the ^1H and ^{13}C NMR spectra reported for the precursor of this dimer, sinapoylagmatine. For this compound the difference between the spectra was also the absence of the two (*trans*)-olefinic protons at [δ_H 7.45 (1H, d, J = 15.65 Hz), δ_H 6.54 (1H, d, J = 15.19 Hz) and and δ_C 140.90, 118.00], and the presence of a singlet at [δ_H 7.92 (2H, s) and δ_C 140.98], and a resonance at δ_C 127.43. Since one proton resonance is lost compared to the monomeric precursor and no other shifts were observed, it was concluded that this dimer is also linked by an 8-8′-linkage, resulting in a symmetrical dimer.

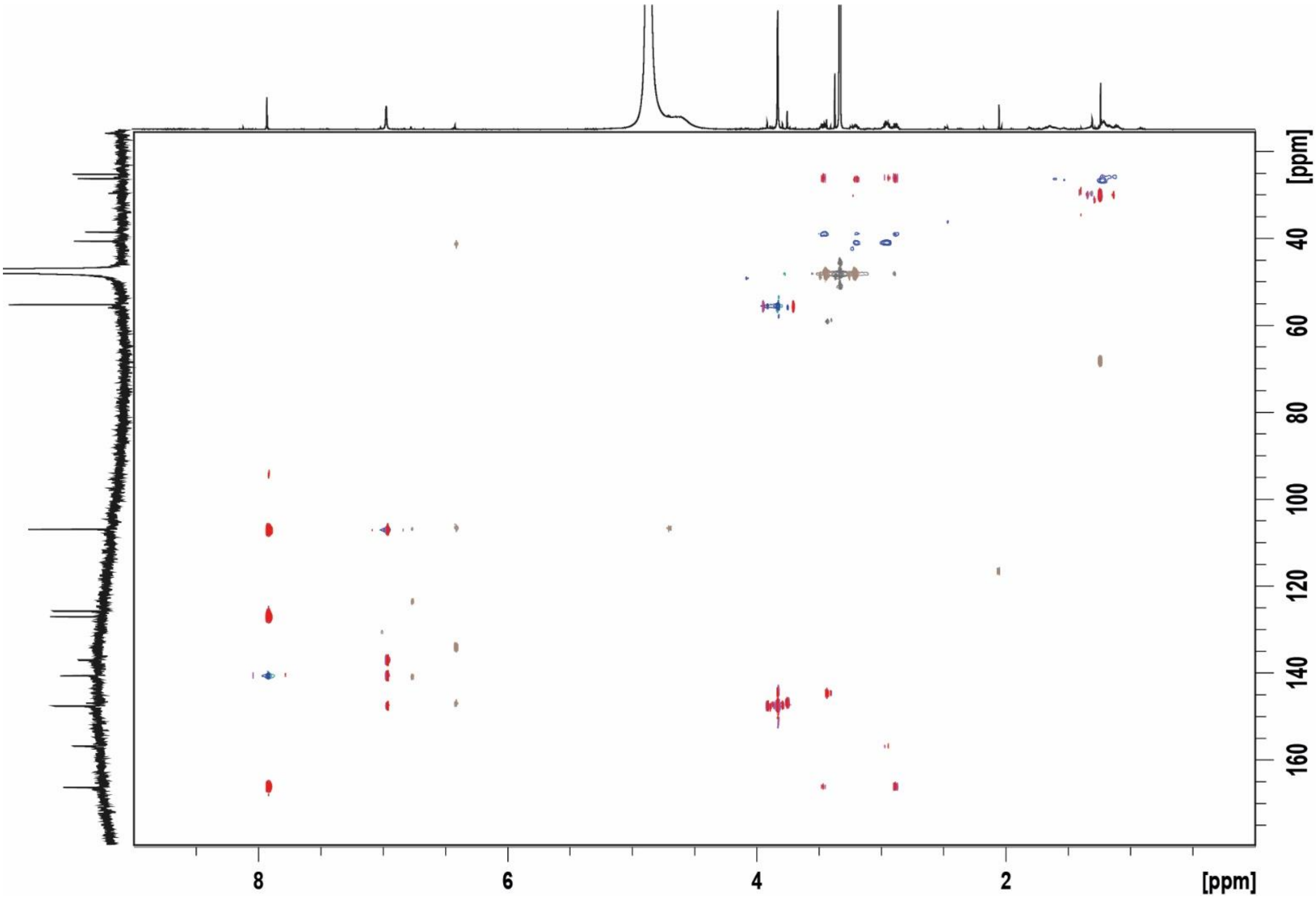

Figure S12. ^1H (600 MHz) and ^{13}C (150 MHz) NMR spectra and HMBC (blue/turquoise) and HSQC (red) correlations for SinAgm-8-8′-SinAgm (27). Impurities in the spectrum are shown in grey (HMBC) and brown (HSQC).

SinAgm-8-8′/9-*N*-7′-DSinAgm (29).

Compound 29 had the molecular formula $C_{32}H_{46}O_8N_8$ as determined by HRMS at *m/z* 336.17892 $[M+2H]^{2+}$ (calculated for $C_{32}H_{48}O_8N_8^{2+}$, 336.17921). The ^1H and ^{13}C NMR spectra (Main text; Table 2) revealed the presence of two agmatine moieties [δ_H 3.24 (2H, m), δ_H 1.57 (4H, m), δ_H 1.37 (4H, m), δ_H 3.20 (4H, m) and δ_C 38.69, 38.80, 25.60, 36.34, 40.63, 157.23], two 1,3,4,5-tetrasubstituted benzene rings at [δ_H 6.77 (2H, s), and δ_C 125.29, 107.49, 148.01, 136.03; δ_H 6.57 (2H, s), and δ_C 130.10, 103.67, 148.53, 137.40], three methines at [δ_H 7.53 (1H, d, J = 2.40 Hz), δ_H 4.64 (n.d., d, J = 3.26 Hz), δ_H 4.00 (1H, m) and δ_C 134.99, 65.67, 53.28], four methoxy groups [δ_H 3.87 (6H, s), δ_H 3.85 (6H, s) and δ_C 55.71, 55.53], and two conjugated carbonyl carbons (δ_C 169.75, 171.80). Since two resonances corresponding to methoxy groups were observed, it was concluded that the ring of both sinapoylagmatine precursors was uninvolved in the coupling, as this would have resulted in loss of symmetry. By HMBC it could be

observed that H-7 correlates with C-8′, however no correlation was found between H-7 and C-7′, indicating a linkage was formed between C-8 and C-8′. Since the typical signal for (*trans*)-olefinic protons was not detected by NMR, it was concluded that the double bond between C-7′ and C-8′ was saturated upon coupling, which hints at a lactam shaped linkage type, since no additional proton was observed for C-7′. In literature this linkage type was reported before for murinamide B, a feruloylagmatine homodimer. The reported ^{1}H and ^{13}C NMR spectra match with the spectra obtained for this compound, with one difference being the presence of an additional methoxy group on both benzene rings.[3] It was therefore concluded this dimer possessed an 8-8′/9-*N*-7′-linkage.

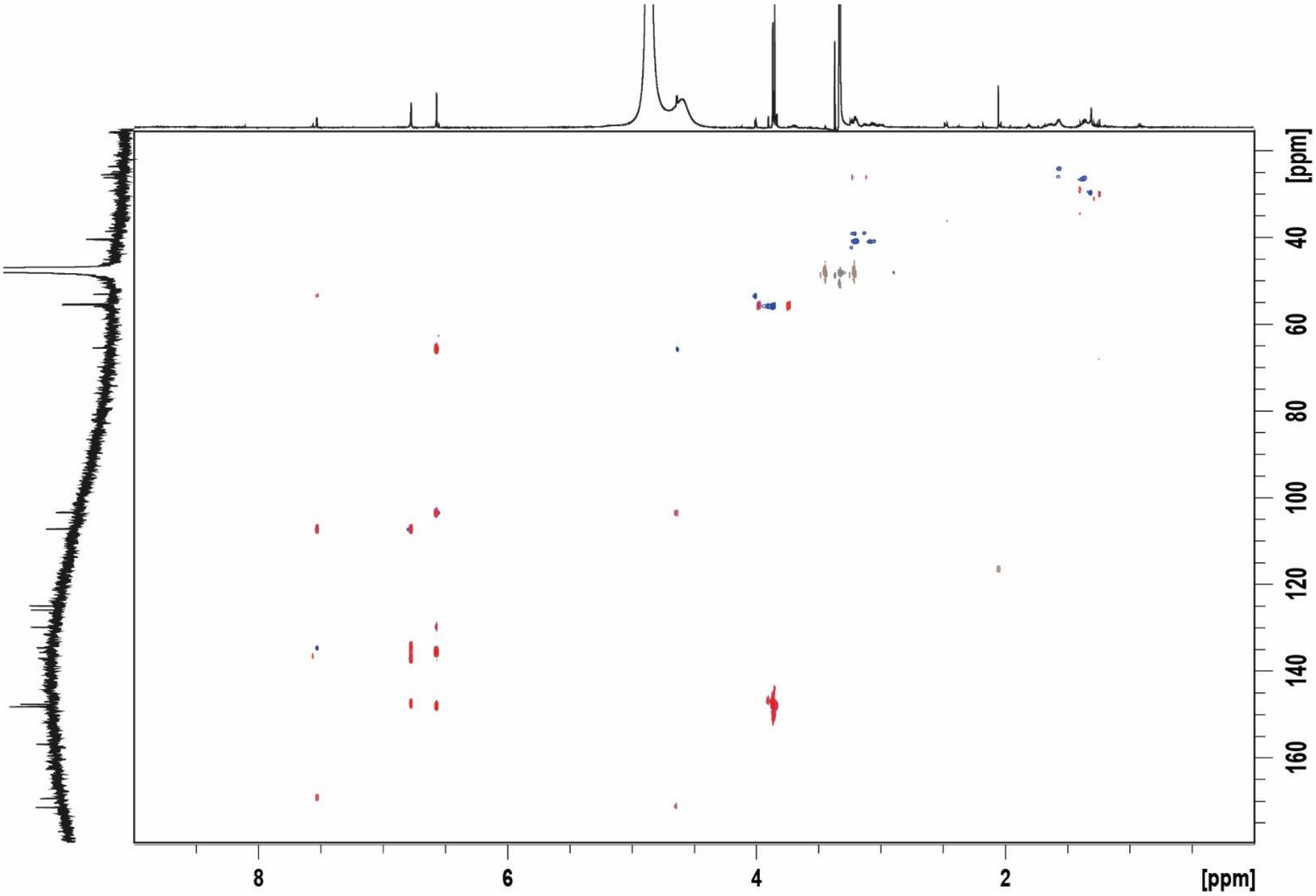

Figure S13. ^{1}H (600 MHz) and ^{13}C (150 MHz) NMR spectra and HMBC (blue/turquoise) and HSQC (red) correlations for SinAgm-8-8′/9-N-7′-DSinAgm (29). Impurities in the spectrum are shown in grey (HMBC) and brown (HSQC).

Annotation of coupling products

Table S1. UHPLC-PDA-ESI-IT-MS data and ESI-FT-MS data for the hydroxycinnamoylagmatine oxidative coupling products after 120 minutes incubation with HRP. Only fragments with a relative abundance above 10% are given. Peak numbers correspond to numbers given in Figure 2 in the manuscript.

UHPLC-PDA-ESI-IT-MS						ESI-FT-MS				
Compound	RT (min)	λ_{max} (nm)	Ionisation	*m/z*	MS^2 (*m/z*) (relative abundance) [a]	Molecular formula	Calculated *m/z*	Observed *m/z*	Error (ppm)	Annotation
CouAgm										
1	5.09	214	$[M+2H]^{2+}$	285	224 (100), 157 (45), 199 (35), 276 (25), 246 (25), 291 (23), 395 (22), 131 (17), 353 (16), 194 (12), 114 (11)	$C_{28}H_{40}O_5N_8$	285.16336	285.16296	−1.40	CouAgm dimer + H_2O
2	6.27	n.d.	$[M+H]^+$	277	260 (100), 235 (41), 114 (24), 217 (20)	$C_{14}H_{20}O_2N_4$	277.16590	277.16565	−0.91	*cis*-CouAgm [c]
3	6.74	318	$[M+2H]^{2+}$	276	421 (100), 131 (90), 234 (79), 211 (76), 114 (72), 404 (53), 246 (53), 362 (46), 230 (30), 361 (28), 422 (26), 255 (23), 267 (20), 157 (20), 534 (18), 405 (15), 231 (15)	$C_{28}H_{38}O_4N_8$	276.15808	276.15771	−1.33	CouAgm-8-8′-DCouAgm [c]
4	8.30	318	$[M+H]^+$	348	331 (100), 185 (50), 218 (25), 168 (18), 114 (18), 306 (12), 202 (12), 288 (12)	$C_{17}H_{25}O_2N_5$	348.20302	348.20270	−0.39	
5	9.55	294	$[M+H]^+$	277	260 (100), 114 (17), 217 (17), 115 (12), 235 (10)	$C_{14}H_{20}O_2N_4$	277.16590	277.16568	−0.81	*trans*-CouAgm [b]
6	11.09	318	$[M+2H]^{2+}$	276	246 (100), 267 (29), 234 (11), 260 (5), 534 (3), 157 (3), 421 (2), 211 (2), 114 (2), 131 (2)	$C_{28}H_{38}O_4N_8$	276.15808	276.15793	0.53	CouAgm-8-8′/9-*N*-7′-DCouAgm [c]
7	11.71	n.d.	$[M+2H]^{2+}$	252	223 (100), 277 (78), 244 (71), 168 (43), 288 (16), 169 (16), 373 (12), 231 (11), 278 (10)	$C_{23}H_{38}O_4N_9$	252.65570	252.65561	−0.09	
8	17.77	318	$[M+2H]^{2+}$	276	267 (100), 246 (77), 421 (22), 211 (19), 234 (13), 420 (13), 114 (12)	$C_{28}H_{38}O_4N_8$	276.15808	276.15820	0.44	
9	19.69	310	$[M+2H]^{2+}$	267	395 (100), 246 (90), 267 (79), 157 (24), 396 (24), 98 (19), 421 (17), 291 (12), 131 (12), 114 (12), 97 (11), 420 (11), 534 (5), 211 (3)	$C_{28}H_{38}O_4N_8$	276.15808	276.15814	0.23	CouAgm-4-*O*-7′/3-8′-DCouAgm [b]
10	19.97	318	$[M+2H]^{2+}$	276	395 (100), 246 (100), 267 (91), 157 (24), 396 (24), 98 (17), 421 (17), 97 (13), 291 (12), 255 (12), 131 (10), 114 (9), 534 (4)	$C_{28}H_{38}O_4N_8$	276.15808	276.15808	−0.01	CouAgm-4-*O*-7′/3-8′-DCouAgm [c]
11	20.50	250	$[M+2H]^{2+}$	276	246 (100), 267 (51), 211 (4), 421 (4), 395 (1), 114 (1)	$C_{28}H_{38}O_4N_8$	276.15808	276.15802	−0.21	CouAgm-4-*O*-8′-CouAgm [c]
FerAgm										
12	10.25	334	$[M+2H]^{2+}$	306	131 (100), 241 (99), 481 (78), 114 (61), 297 (41), 157 (39), 464 (36), 276 (31), 323 (31), 422 (29), 227 (27), 482 (26), 285 (26), 179 (25), 298 (22), 421 (17), 594 (16), 436 (14), 465 (11), 351 (10)	$C_{30}H_{42}O_6N_8$	306.16864	306.16864	−0.20	FerAgm-8-8′-FerAgm [b]
13	10.38	334	$[M+2H]^{2+}$	306	131 (100), 481 (60), 464 (58), 241 (42), 114 (39), 297 (37), 157 (29), 227 (25), 422 (23), 323 (22), 436 (21), 594 (17), 285 (17), 465 (17), 340 (16), 482 (16), 298 (16), 276 (12), 439 (12), 331 (10)	$C_{30}H_{42}O_6N_8$	306.16864	306.16840	−0.79	FerAgm-2-7′/8-8′-DFerAgm [b]
14	12.25	318	$[M+H]^+$	307	290 (100), 177 (22), 114 (18), 247 (14)	$C_{15}H_{22}O_3N_4$	307.17647	307.17633	−0.45	*trans*-FerAgm [b]

15	13.27	n.d.	$[M+2H]^{2+}$	267	307 (100), 238 (66), 259 (55), 168 (45), 228 (33), 308 (17), 169 (16)	$C_{24}H_{40}O_5N_9$	267.99098	267.66092	−0.03	
16	14.07	n.d.	$[M+2H]^{2+}$	306	276 (100), 297 (32), 285 (11), 131 (10), 157 (7), 481 (6), 241 (6), 114 (5), 594 (4), 290 (4)	$C_{30}H_{42}O_6N_8$	306.16864	306.16861	−0.10	FerAgm-8-8′/9-*N*-7′-DFerAgm [c]
17	16.55	n.d.	$[M+2H]^{2+}$	306	481 (100), 131 (86), 114 (53), 157 (53), 331 (45), 297 (38), 298 (30), 265 (26), 464 (26), 482 (25), 340 (23), 422 (22), 323 (15), 227 (14), 285 (14), 285 (14), 594 (13), 271 (12), 264 (12), 201 (11)	$C_{30}H_{42}O_6N_8$	306.16864	306.16849	−0.50	
18	21.06	322	$[M+2H]^{2+}$	306	455 (100), 297 (55), 276 (49), 157 (44), 481 (27), 456 (27), 131 (16), 351 (12), 114 (10)	$C_{30}H_{42}O_6N_8$	306.16864	306.16870	0.19	FerAgm-4-*O*-7′/3-8′-DFerAgm [b]
19	21.19	330	$[M+2H]^{2+}$	306	455 (100), 297 (60), 276 (58), 157 (47), 449 (42), 131 (30), 286 (26), 281 (25), 393 (24), 456 (21), 363 (21), 114 (14), 265 (11), 351 (11)	$C_{30}H_{42}O_6N_8$	306.16864	306.16858	−0.20	
20	22.01	322	$[M+2H]^{2+}$	306	455 (100), 297 (53), 276 (51), 157 (44), 456 (25), 481 (24), 131 (15), 351 (9), 114 (8)	$C_{30}H_{42}O_6N_8$	306.16864	306.16855	−0.30	FerAgm-4-*O*-7′/3-8′-DFerAgm [c]
21	23.26	326	$[M+2H]^{2+}$	306	276 (100), 297 (48), 289 (14), 481 (4), 241 (2), 455 (2), 114 (1), 290 (1)	$C_{30}H_{42}O_6N_8$	306.16864	306.16855	−0.30	FerAgm-4-*O*-8′-FerAgm [b]
					SinAgm					
22	5.12	n.d.	$[M+2H]^{2+}$	345	306 (100), 336 (66), 327 (43), 315 (21), 361 (20), 254 (19), 157 (12), 131 (12)	$C_{32}H_{50}O_9N_8$	345.18449	345.18427	−0.63	
23	6.48	n.d.	$[M+2H]^{2+}$	345	336 (100), 306 (66), 254 (45), 327 (30), 361 (13), 224 (12), 315 (11)	$C_{32}H_{50}O_9N_8$	345.18449	345.18427	−0.64	
24	7.55	n.d.	$[M+2H]^{2+}$	345	336 (100), 319 (78), 353 (45), 157 (33), 515 (32), 320 (15)	$C_{32}H_{50}O_9N_8$	345.18449	345.18433	−0.46	
25	8.87	330	$[M+2H]^{2+}$	336	131 (100), 361 (99), 157 (64), 328 (60), 383 (57), 306 (57), 327 (56), 370 (54), 271 (52), 541 (45), 315 (43), 114 (35), 344 (29), 301 (21), 496 (19), 362 (18), 181 (17), 654 (15), 524 (15), 542 (14)	$C_{32}H_{46}O_8N_8$	336.17921	336.17923	0.07	SinAgm-2-7′/8-8′-SinAgm [b]
26	10.76	314	$[M+2H]^{2+}$	336	541 (100), 361 (65), 131 (59), 327 (57), 157 (41), 387 (35), 114 (32), 542 (27), 370 (27), 328 (25), 344 (21), 411 (18), 524 (15), 155 (13), 362 (11), 139 (11), 301 (11), 181 (10)	$C_{32}H_{46}O_8N_8$	336.17921	336.17899	−0.65	
27	11.21	338	$[M+2H]^{2+}$	336	271 (100), 131 (81), 315 (79), 306 (78), 541 (71), 411 (69), 383 (50), 157 (42), 328 (36), 327 (34), 114 (31), 542 (23) 524 (19), 344 (18), 361 (18), 412 (17), 370 (16), 387 (15), 231 (13)	$C_{32}H_{46}O_8N_8$	336.17921	336.17899	−0.65	SinAgm-8-8′-SinAgm [b]
28	13.55	n.d.	$[M+2H]^{2+}$	336	306 (100)	$C_{32}H_{46}O_8N_8$	336.17921	336.17892	−0.85	
29	13.72	330	$[M+2H]^{2+}$	336	306 (100), 327 (30), 315 (14), 157 (9) 131 (8), 320 (6), 114 (5), 271 (5), 541 (4), 654 (3)	$C_{32}H_{46}O_8N_8$	336.17921	336.17905	−0.47	SinAgm-8-8′/9-*N*-7′-DSinAgm [b]
30	14.18	322	$[M+H]^{+}$	337	320 (100), 207 (55), 114 (18), 303 (14), 277 (12), 115 (10)	$C_{16}H_{24}O_4N_4$	337.18703	337.18674	−0.87	*trans*-SinAgm [b]

[a] All fragments with a relative abundance of 10 % or higher and fragments linked to the fragmentation patterns (Main text; Figure 4) are reported, [b] Identified by NMR, [c] Identified based on fragmentation pattern, n.d. = not detected

MS^2 spectra

Table S2. ESI-IT-MS^2 CID spectra for the different linkage types per monomer.

Linkage	CouAgm MS^2	FerAgm MS^2	SinAgm MS^2
4-*O*-7′/3-8′	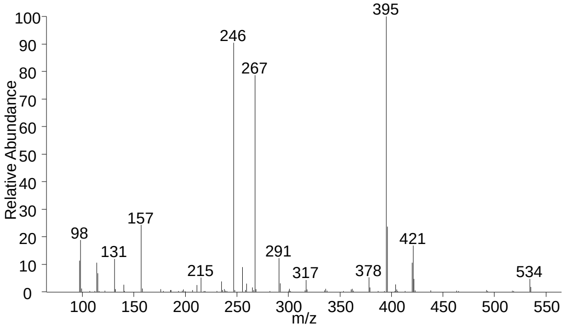	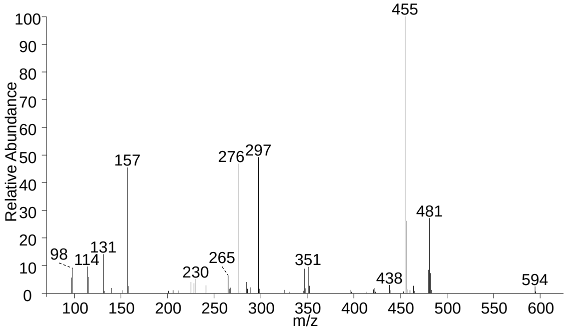	
8-8′/9-*N*-7′	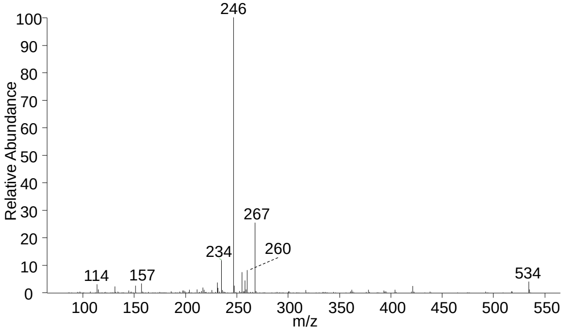	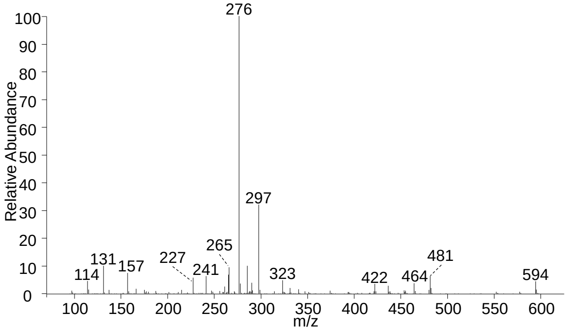	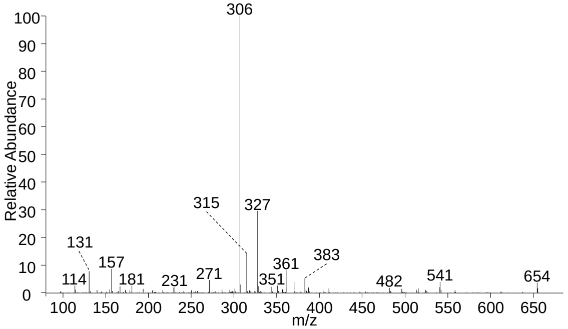

Linkage	CouAgm MS^2	FerAgm MS^2	SinAgm MS^2

2-7′/8-8′

FerAgm MS^2

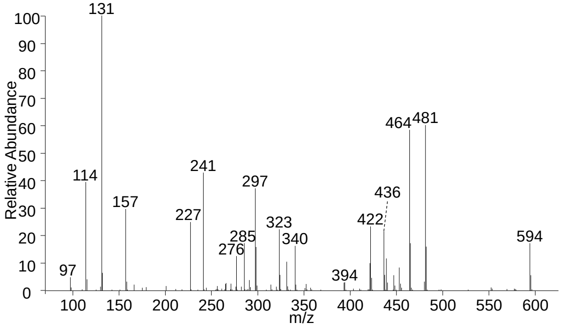

SinAgm MS^2

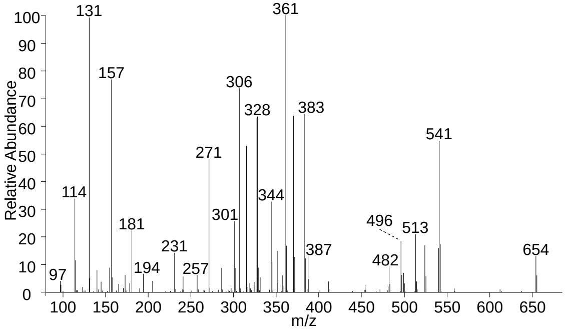

8-8′

CouAgm MS^2

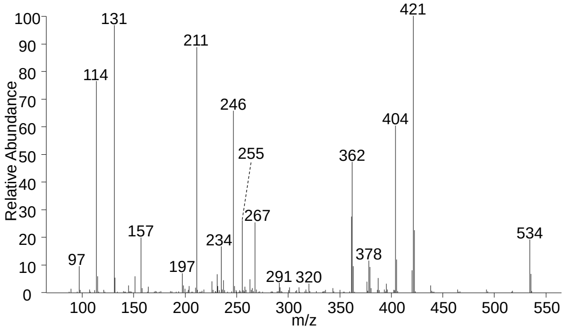

FerAgm MS^2

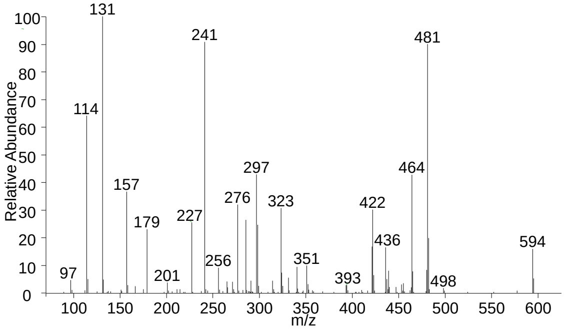

SinAgm MS^2

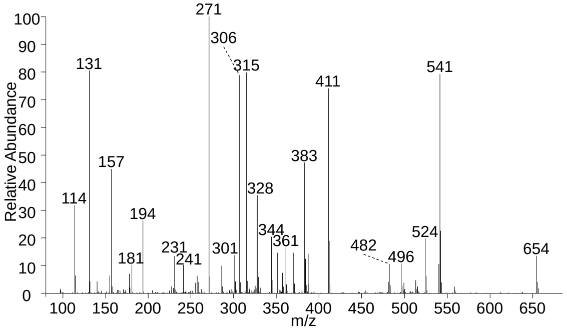

4-*O*-8′

CouAgm MS^2

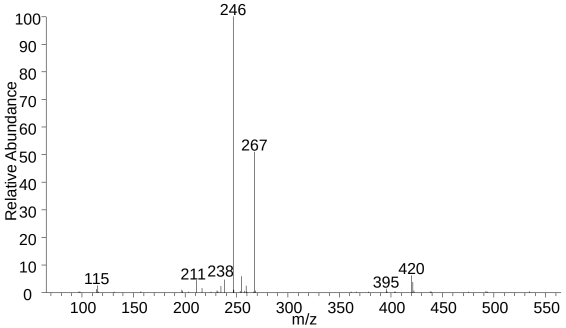

FerAgm MS^2

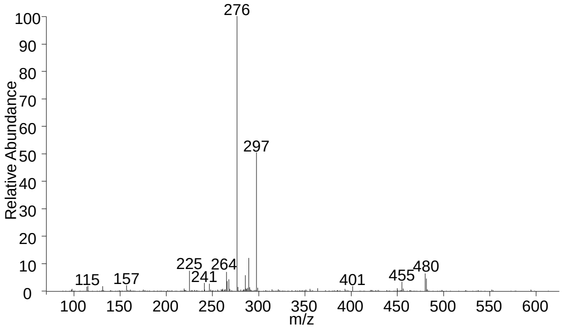

Figure S14. Identification of the main fragments in ESI-IT-MS² CID spectra by determination of the elemental composition of the fragments by ESI-FT-MS². Numbers between brackets correspond to the mass error in ppm.

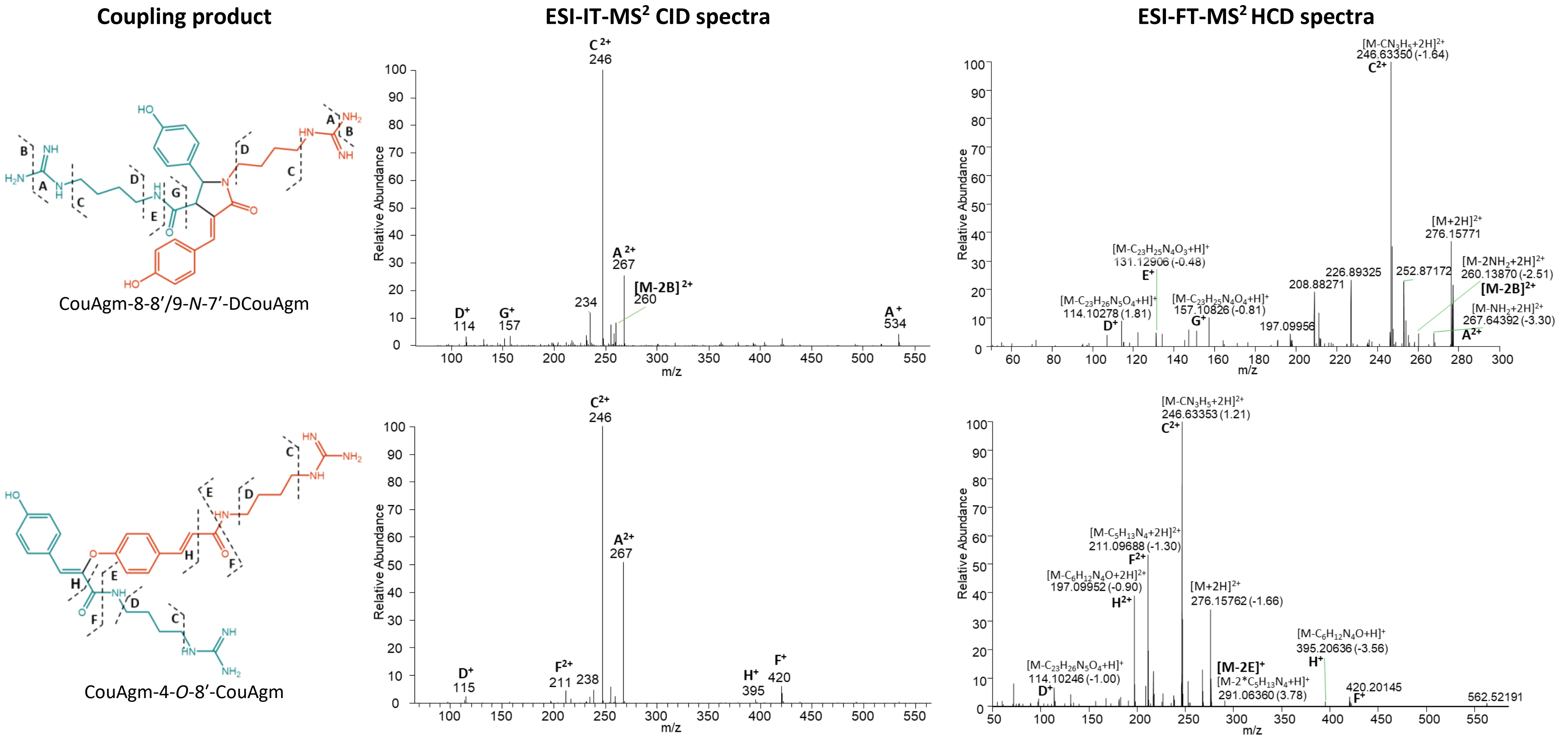

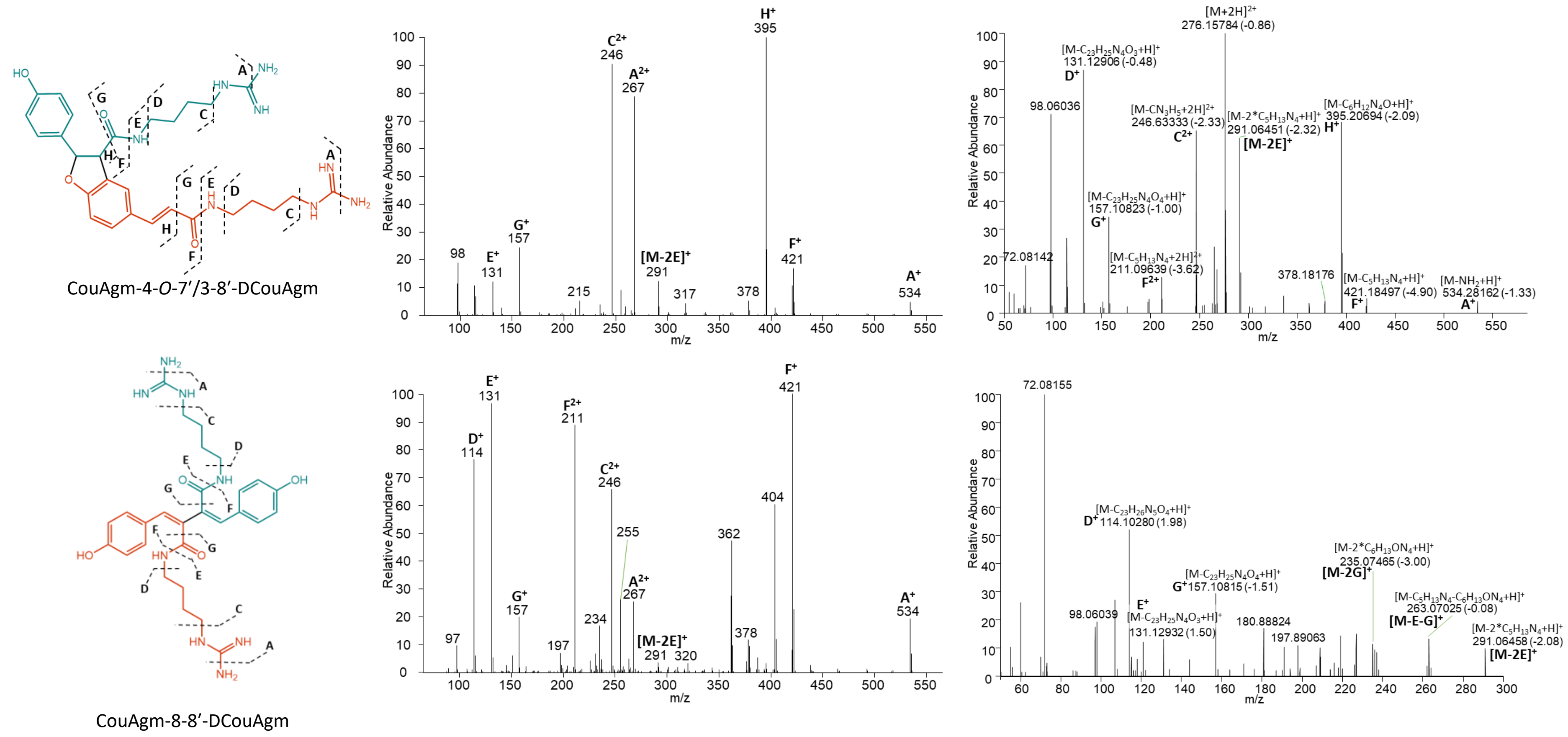

CouAgm-4-O-7′/3-8′-DCouAgm
CouAgm-8-8′-DCouAgm
Relative Abundance
m/z
H+ 395
C2+ 246
A2+ 267
G+ 157
E+ 131
98
215
[M-2E]+ 291
317
378
F+ 421
A+ 534
[M+2H]2+ 276.15784 (-0.86)
[M-C23H25N4O3+H]+ 131.12906 (-0.48) D+
98.06036
[M-CN3H5+2H]2+ 246.63333 (-2.33) C2+
[M-2*C5H13N4+H]+ 291.06451 (-2.32) [M-2E]+
[M-C6H12N4O+H]+ 395.20694 (-2.09) H+
[M-C23H25N4O4+H]+ 157.10823 (-1.00) G+
72.08142
[M-C5H13N4+2H]2+ 211.09639 (-3.62) F2+
378.18176
[M-C5H13N4+H]+ 421.18497 (-4.90) F+
[M-NH2+H]+ 534.28162 (-1.33) A+
E+ 131
D+ 114
F2+ 211
C2+ 246
255
A2+ 267
234
97
197
[M-2E]+ 291
320
362
378
404
F+ 421
A+ 534
72.08155
[M-C23H26N5O4+H]+ D+ 114.10280 (1.98)
[M-C23H25N4O4+H]+ G+ 157.10815 (-1.51)
98.06039
E+ [M-C23H25N4O3+H]+ 131.12932 (1.50)
180.88824
197.89063
[M-2*C6H13ON4+H]+ 235.07465 (-3.00) [M-2G]+
[M-C5H13N4-C6H13ON4+H]+ 263.07025 (-0.08) [M-E-G]+
[M-2*C5H13N4+H]+ 291.06458 (-2.08) [M-2E]+

Effect of pH on oxidative coupling

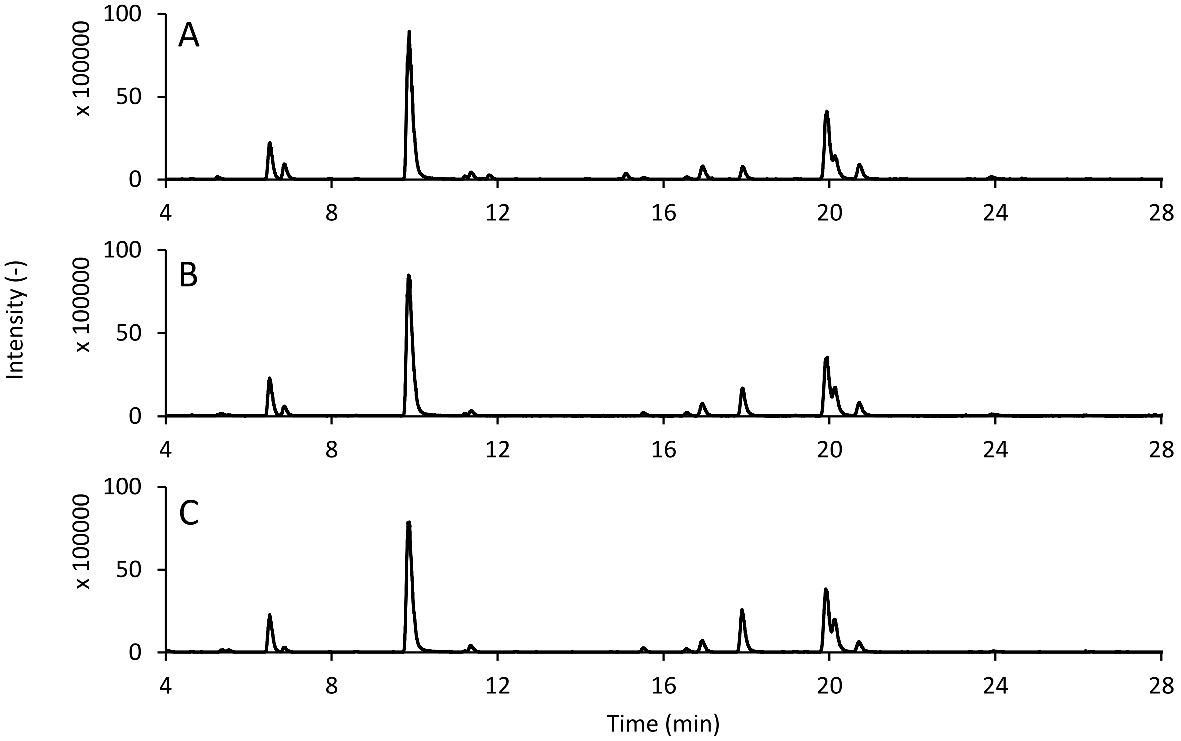

Figure S15. RP-UHPLC-PDA-IT-MS base peak chromatograms (m/z 250-1500) in positive mode after 120 minutes incubation of CouAgm with HRP and H_2O_2 at pH 5 (A), pH 7 (B), and pH 8.5 (C).

MALDI-TOF-MS

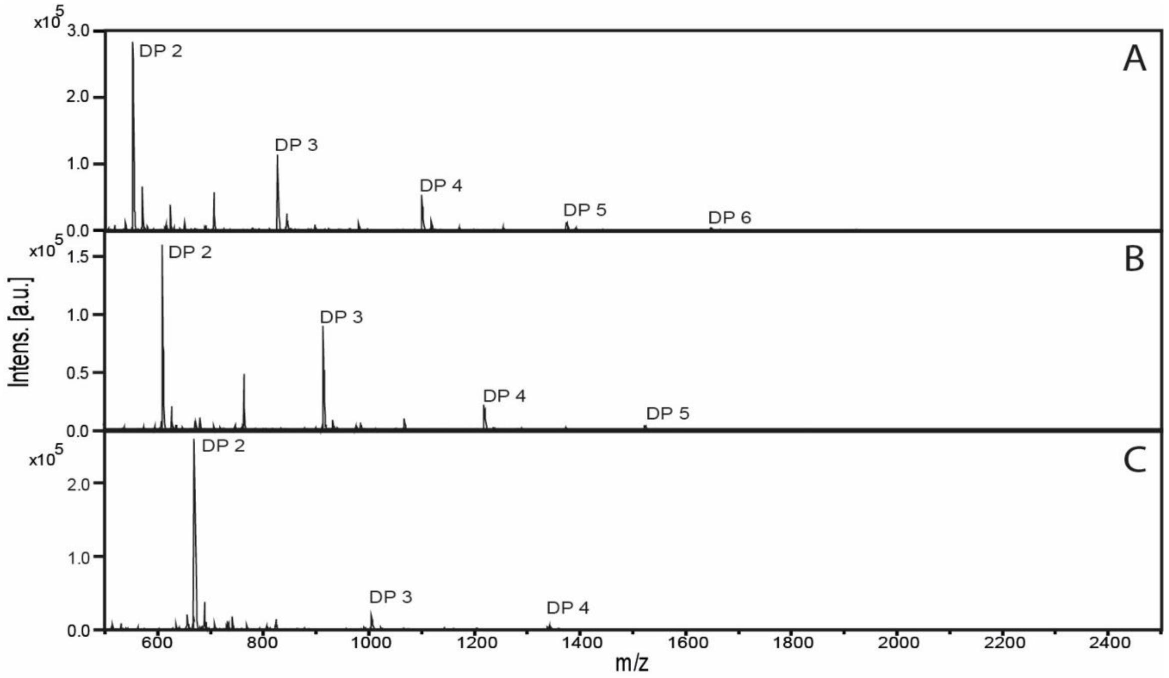

Figure S16. MALDI-TOF-MS spectra of (A) CouAgm, (B) FerAgm, and (C) SinAgm incubated with HRP at t=120 min. DP = degree of polymerisation.

References

1. Jin, S.; Yoshida, M., Antifungal compound, feruloylagmatine, induced in winter wheat exposed to a low temperature. *Biosci., Biotechnol., Biochem.* **2000,** *64* (8), 1614-1617.
2. Sakakibara, I.; Ikeya, Y.; Hayashi, K.; Okada, M.; Maruno, M., Three acyclic bis-phenylpropane lignanamides from fruits of *Cannabis sativa*. *Phytochemistry* **1995,** *38* (4), 1003-1007.
3. Ube, N.; Nishizaka, M.; Ichiyanagi, T.; Ueno, K.; Taketa, S.; Ishihara, A., Evolutionary changes in defensive specialized metabolism in the genus *Hordeum*. *Phytochemistry* **2017,** *141*, 1-10.
4. Spreng, S.; Hofmann, T., Activity-guided identification of in vitro antioxidants in beer. *J. Agric. Food. Chem.* **2018,** *66* (3), 720-731.
5. Zhang, B.; Huang, R.; Hua, J.; Liang, H.; Pan, Y.; Dai, L.; Liang, D.; Wang, H., Antitumor lignanamides from the aerial parts of *Corydalis saxicola*. *Phytomedicine* **2016,** *23* (13), 1599-1609.
